# Deep Cerebellar Transcranial Direct Current Stimulation of the Dentate Nucleus to Facilitate Standing Balance in Chronic Stroke Survivors—A Pilot Study

**DOI:** 10.3390/brainsci10020094

**Published:** 2020-02-10

**Authors:** Zeynab Rezaee, Surbhi Kaura, Dhaval Solanki, Adyasha Dash, M V Padma Srivastava, Uttama Lahiri, Anirban Dutta

**Affiliations:** 1 Department of Biomedical Engineering, University at Buffalo, The State University of New York, Buffalo, NY 14260, USA; zeynabre@buffalo.edu; 2 All India Institute of Medical Sciences, New Delhi 110029, India; physiosurbhi28@gmail.com (S.K.); vasanthapadma123@gmail.com (M.V.P.S.); 3 Indian Institute of Technology Gandhinagar, Palaj 382355, India; dhaval.solanki@iitgn.ac.in (D.S.); adyasha.dash@iitgn.ac.in (A.D.); uttamalahiri@iitgn.ac.in (U.L.)

**Keywords:** cerebellar transcranial direct current stimulation, dentate nucleus, computational modeling

## Abstract

Objective: Cerebrovascular accidents are the second leading cause of death and the third leading cause of disability worldwide. We hypothesized that cerebellar transcranial direct current stimulation (ctDCS) of the dentate nuclei and the lower-limb representations in the cerebellum can improve functional reach during standing balance in chronic (>6 months’ post-stroke) stroke survivors. Materials and Methods: Magnetic resonance imaging (MRI) based subject-specific electric field was computed across a convenience sample of 10 male chronic (>6 months) stroke survivors and one healthy MRI template to find an optimal bipolar bilateral ctDCS montage to target dentate nuclei and lower-limb representations (lobules VII–IX). Then, in a repeated-measure crossover study on a subset of 5 stroke survivors, we compared 15 min of 2 mA ctDCS based on the effects on successful functional reach (%) during standing balance task. Three-way ANOVA investigated the factors of interest– brain regions, montages, stroke participants, and their interactions. Results: “One-size-fits-all” bipolar ctDCS montage for the clinical study was found to be PO9h–PO10h for dentate nuclei and Exx7–Exx8 for lobules VII–IX with the contralesional anode. PO9h–PO10h ctDCS performed significantly (alpha = 0.05) better in facilitating successful functional reach (%) when compared to Exx7–Exx8 ctDCS. Furthermore, a linear relationship between successful functional reach (%) and electric field strength was found where PO9h–PO10h montage resulted in a significantly (alpha = 0.05) higher electric field strength when compared to Exx7–Exx8 montage for the same 2 mA current. Conclusion: We presented a rational neuroimaging based approach to optimize deep ctDCS of the dentate nuclei and lower limb representations in the cerebellum for post-stroke balance rehabilitation. However, this promising pilot study was limited by “one-size-fits-all” bipolar ctDCS montage as well as a small sample size.

## 1. Introduction

Cerebrovascular accidents (stroke) are the second leading cause of death and the third leading cause of disability worldwide (Global Health Estimates. Geneva, Switzerland: World Health Organization; 2012). Worldwide, 70% of strokes and 87% of both stroke-related deaths and disability-adjusted life years occur in low- and middle-income countries (LMIC) [1]. While the incidence of stroke is decreasing in the developed world [1], the incidence is increasing in India, an LMIC, due to demographic transition and a rapid shift in the socio-economic milieu. The estimated adjusted prevalence rate of stroke ranges from 84–262/100,000 in rural and 334–424/100,000 in urban India, and the incidence rate is 119–145/100,000 based on the recent population-based studies [2]. Thus, stroke constitutes a substantial socioeconomic burden on the patients, caregivers, and society in India [3,4]. The scarcity of trained rehabilitation clinicians, as well as the cost of clinic-based rehabilitation programs, can deter stroke survivors from undergoing regular post-stroke rehabilitation leading to further decline in their health conditions. Given the high prevalence and incidence of stroke in India, there is a need to investigate low-cost neurotechnologies to facilitate early post-stroke rehabilitation.

Early task-specific rehabilitation after stroke may drive functionally relevant beneficial neuroplastic changes in the brain where neuroplasticity is the ability of the central nervous system to respond to intrinsic or extrinsic stimuli by reorganizing its structure, function, and connections. Recent clinical studies in the USA on invasive deep brain stimulation of the cerebellum for post-stroke motor rehabilitation are based on the extensive reciprocal connectivity between the dentate nucleus and the wide swaths of cerebral cortex via the dentatothalamocortical and corticopontocerebellar tracts [5]. Dentate is a promising target for brain stimulation since it remains mostly unaffected by lesions [6], and deep brain stimulation of the cerebellum is proposed to ameliorate the known limitations to motor rehabilitation imposed by crossed cerebellar diaschisis. Here, the improvements in motor function are found paralleled by increased expression of markers of synaptic plasticity, synaptogenesis, and neurogenesis in the perilesional cortex. In this study, we investigated a low-cost non-invasive brain stimulation (NIBS) approach to the cerebellum [7,8] based on cerebellar transcranial direct current stimulation (ctDCS). ctDCS has been found to be a promising method to facilitate cerebellar functions [9] where it can improve locomotor adaptation [10] as well as postural recovery from disturbance by Achilles tendon vibration [11]. The conventional ctDCS electrode montages [12] most likely produce their effects by polarizing Purkinje cells [13], and its therapeutic effects are an adjunct to motor training [8]. In this study, we aimed to directly target the dentate nucleus with ctDCS which is the largest and most lateral of the four deep cerebellar nuclei and is known to be involved in planning and executing voluntary movements [14]. In fact, the dentate nucleus can affect motor as well as cognitive function [12] due to extensive reciprocal connectivity between the dentate nucleus and the wide swaths of the cerebral cortex. Since dentate receives proprioceptive information from the spinocerebellar tract via the inferior cerebellar peduncle and also receives planning and initiating voluntary movement-related information from the premotor and supplementary motor cortices so it can perform error computations (comparator function) relevant to maintain timing, balance, and equilibrium. Therefore, we postulate that subthreshold stimulation of the dentate nucleus can help generate appropriately timed burst activity [15] during standing balance functional reach tasks (FRT) using a human-machine interface (HMI) [16]. Our prior work on ctDCS optimization [7] showed that highly conductive cerebrospinal fluid can provide a path for the stimulation current to reach the depths of the vermis, and since the dentate nucleus is directly adjacent to the vermis and the roof of the fourth ventricle bilaterally so we aimed to directly target dentate nucleus with ctDCS to facilitate cued weight-shifting in chronic stroke survivors. Here, patient selection may be crucial for ctDCS as an adjunct to post-stroke balance rehabilitation, e.g., in patients where the cerebellum is without lesion and the cerebello-cerebral connectivity is intact, since stroke is a heterogeneous disease with different mechanisms and etiologies.

While non-invasive brain stimulation (NIBS) techniques, including tDCS, are increasingly used for the modulation of corticospinal excitability in humans by passing low electric currents through the brain, its treatment effects are rather inconsistent across studies [17,18]. Besides usability issues, one of the important factors contributing to the inconsistency is the lack of expertise in individualizing tDCS [17,18]. For example, ctDCS has shown promise in improving standing balance performance in small studies with fifteen patients with chronic stroke (>6 months post-stroke) [19] where exploration of optimal timing, dose, and the relation between qualitative parameters and clinical improvements are needed [19]. A recent study [20] showed that multiple sessions (three sessions of 20 min per week for two weeks) of simultaneous postural training with bilateral anodal ctDCS (not postural training or bilateral anodal ctDCS alone) was necessary to deliver therapeutic effects in older adults with high fall risk. However, due to heterogeneous brain lesions in stroke, subject-specific optimization of the ctDCS electric field using Magnetic Resonance Imaging (MRI) data and a computational pipeline [7] is important. We have shown that different ctDCS electrode montages affect different parts or lobules of the cerebellum [7,21], however, the related behavioral effects could not be determined in our prior works in the absence of patient data. In the current pilot study, we tested the usability and feasibility of a bipolar bilateral ctDCS in chronic (>6 months) stroke survivors where a group-averaged optimal bipolar bilateral ctDCS montage was developed based on subject-specific optimization across post-stroke participant MRI as well as based on a healthy MRI template. Usability testing with well-defined neuroimaging based customization outside of laboratory setting is necessary for strengthening remote patient care and monitoring for chronic stroke conditions. Here, heterogeneously lesioned brain regions after stroke present a challenge because of the alterations of current flow, which may require the development of individualized ctDCS electrode montage based on neuroimaging [7]. In this study, we selected stroke survivors with cerebral lesions but with an intact cerebellum so that the ctDCS electric field effects can be delivered via intact cerebellum [8]. We first optimized ctDCS with a whole head electrode montage using an age-appropriate human brain MRI template for the age-group of 55 to 59 years (https://jerlab.sc.edu/projects/neurodevelopmental-mri-database/) to select a reduced set of electrodes that were then used to optimize bipolar bilateral ctDCS montage based on the MRIs from our post-stroke participants.

Our computational modeling pipeline [7] and FRT evaluated a bipolar bilateral ctDCS montage to maximally (with electric field strength) target bilateral dentate nuclei (postulated to affect motor as well as cognitive function [12]) versus one optimized to uniformly target the leg area of the cerebellum (i.e., comparable electric field in X, Y, Z directions across lobules VII–IX) [7]. User experience due to 2 mA bipolar ctDCS were monitored by asking participants whether they experienced any of the following symptoms since the preceding treatment: scalp pain, headache, neck pain, dental pain, tingling, nausea, itching, burning sensation, skin redness, open lesion on skin, abnormal sleep, anxiety, difficulty concentrating, dizziness, impaired memory, altered mood, altered balance, impaired use of the unaffected side, or any other problem [22].

## 2. Materials and Methods

### 2.1. Experimental Setup and Study Design

**Figure 1:**
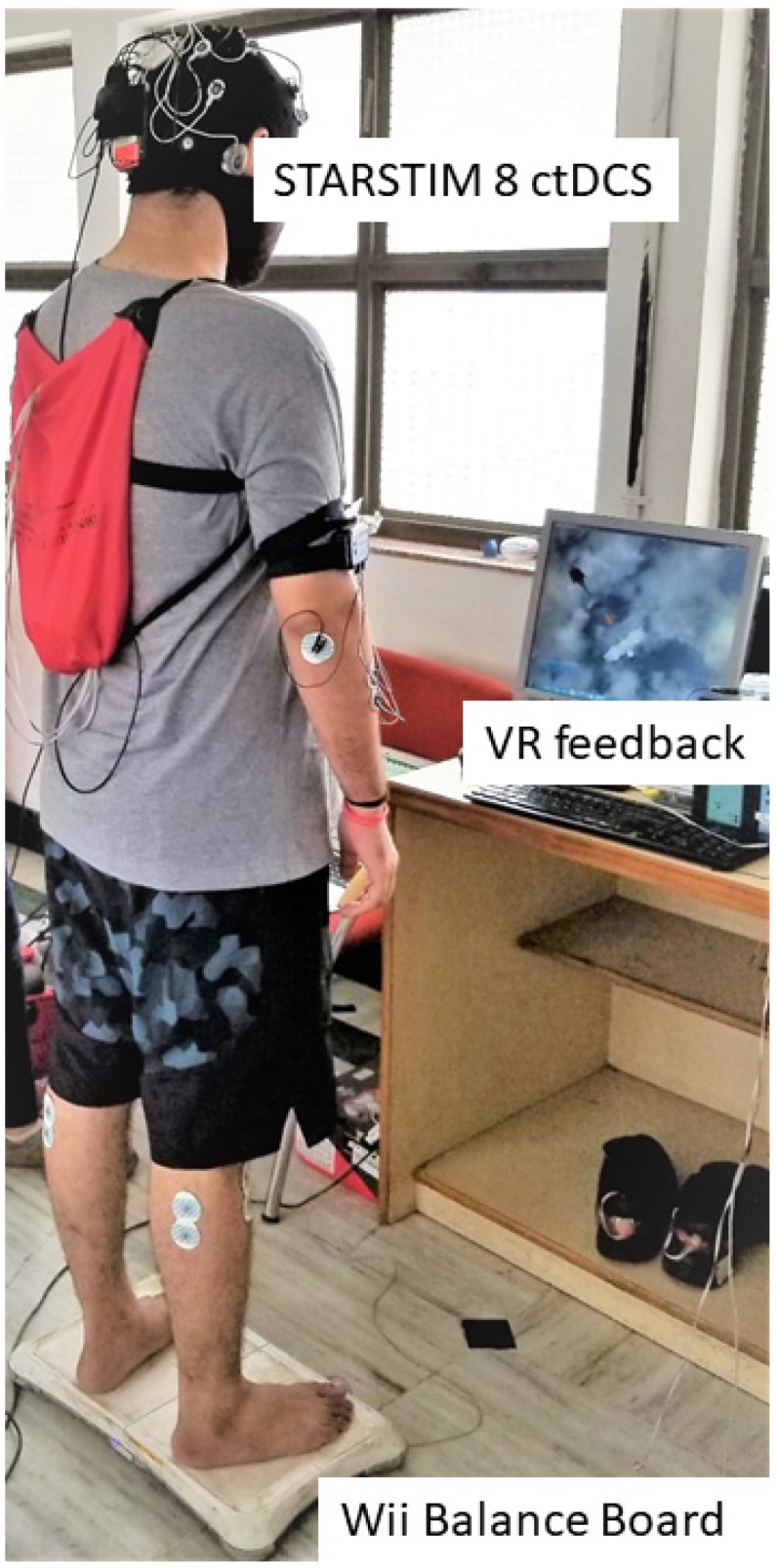
Portable experimental setup for the clinical study consisting of the Wii Balance Board to measure the center of pressure (CoP), a small form factor desktop PC with monitor for the virtual reality (VR) balance testing and training based on CoP. And the wireless STARSTIM 8 simulator for cerebellar transcranial direct current stimulation (ctDCS).

Figure 1 shows the portable experimental setup for the clinical study in a low resource setting. The experimental setup consisted of a portable Wii Balance Board (WiiBB), a small form factor desktop PC with monitor for the VR-based balance training platform [23], and wireless STARSTIM 8 stimulator (Neuroelectrics, Spain) for cerebellar transcranial direct current stimulation (ctDCS). Based on Van de Winckel [22], the capacity building for the clinical study included: (1) ctDCS treatment design and supervision of ctDCS to facilitate VR-based FRT; (2) assessment of the stroke survivor’s capability to participate in VR-based FRT; (3) ongoing training procedures and materials including assessments of the stroke survivor using VR-based FRT; (4) simple and fail-safe electrode placement technique using a neoprene cap; (5) dose estimation based on computational modeling; (6) quantifying compliance by the rehabilitation specialist at the site (ctDCS device preparation, electrode saturation/placement, stimulation protocol), with corresponding corrective steps as required; (7) monitoring for treatment-emergent adverse effects; (8) procedures for discontinuation of a session or study participation including emergency failsafe procedures tailored to the treatment population’s level of need. The subject-specific ctDCS dose (Guideline 5) was simulated by the first author and confirmed by the last author based on our computational modeling pipeline [7] to reduce potential adverse events due to electric field spillover to the lesioned cerebral regions.

A convenience sample of ten male chronic (>6 months post-stroke) stroke subjects participated in the subject-specific MRI-based computational modeling [7]. Based on the assessment of the stroke survivor’s capability to participate in the VR-based FRT, only five subjects (listed in Table 1) completed both the interventions of ctDCS with pre/post FRT in a repeated-measure single-blind counterbalanced study. Two subjects could only attend a single intervention due to scheduling conflicts. Written informed consent was obtained from each participant, and the research protocol for this study was approved by the All India Institute of Medical Sciences New Delhi, India Institutional Review Board (IEC-129/07.04.2017).

**Figure 2:**
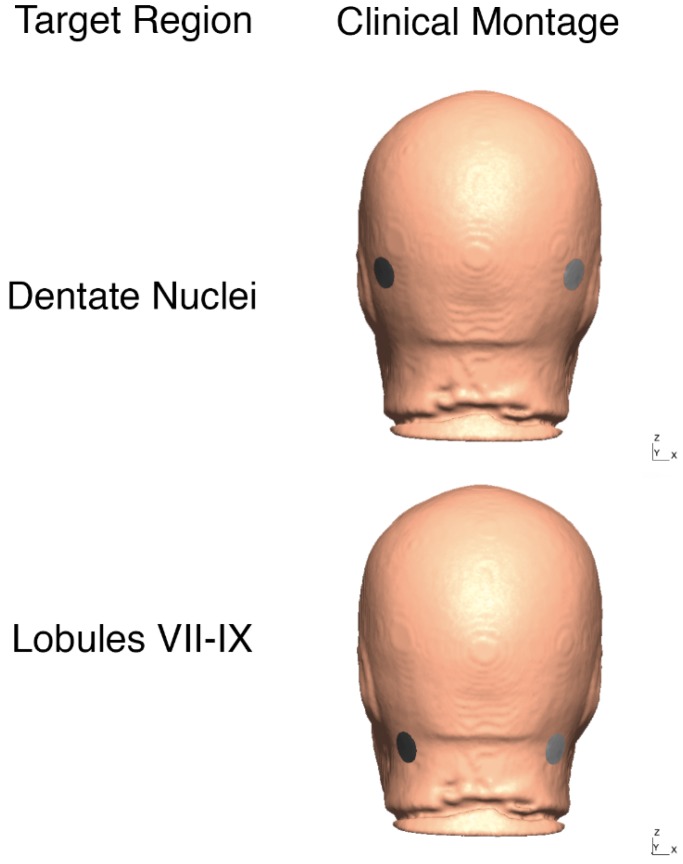
Clinical bilateral ctDCS montages used for FRT balance study is shown on the head model from 55 to 59 years age-group MRI template. Top panel: bipolar at PO9h-PO10h, Bottom panel: bipolar at Exx7–Exx8.

During each session, chronic stroke participants performed FRT for 10 min for a baseline measure of the CoP target reach performance. During FRT [24,25,26], the participant was offered a VR-based target stimulus where CoP (from WiiBB) was mapped to the dynamic position of a VR cursor object that could be modulated to reach the VR target object using weight-shifts on the WiiBB. The details of the VR-based balance training platform are provided in Verma et al. [23]. Briefly, the subject needs to reach a peripheral VR target in the front or to the sides by weight shifting the CoP within a fixed time. Following baseline measure of the CoP target reach performance, 15 min of 2 mA bilateral ctDCS delivered using either of the two bipolar montages with a 1cm radius circular contralesional anode. The electrode locations were based on the Realistic volumetric Approach to Simulate Transcranial Electric Stimulation (ROAST) toolbox [27] and “unambiguously illustrated (UI) 10/5 system” [28] (illustrated in the head model in Figure 2), 1. PO9h–PO10h, and 2. Exx7–Exx8. Further details on computational modeling are provided in Section 2.3. FRT for 10 min was repeated after ctDCS intervention for a post-intervention measure of the CoP target reach performance.

### 2.2. Data Acquisition and Head Modeling

The head model was constructed using subject-specific MRIs from ten male stroke survivors (see Table 1) collected based on the research protocol which was approved by the All India Institute of Medical Sciences (AIIMS), New Delhi, India Institutional Review Board. All subjects were screened for their eligibility to be included in this neuroimaging study by the Department of Nuclear Magnetic Resonance (NMR) at AIIMS. MRI was performed by a 3 Tesla (Achieva or Ingenia, Philips Medical Systems) MR unit using a sixteen multichannel receiver head coil. The MR sequence consists following parameter: MPRAGE, 192 slices, matrix size = 240 × 220, Flip/Flop angle = 8/0, TR/TE = 8.0/3.7 [29,30].

**Figure A1:**
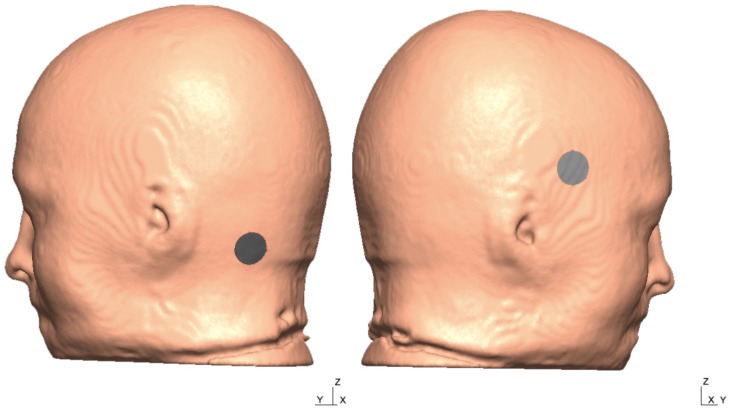
The head model from the 55–59 years old MRI template is shown below with two tentative electrode locations and the X, Y, and the Z-direction of the global coordinate system.

For computational modeling and whole head electrode optimization (details provided in Section 2.3), an age-appropriate averaged (*n* = 73) human brain MRI template for the 55 to 59 years age-group was obtained online at https://jerlab.sc.edu/projects/neurodevelopmental-mri-database/ with the permission of Dr. John Richards. The subjects were all normal healthy adults with no history of neurological or psychiatric illness, head trauma with loss of consciousness, or current or past use of psycho-stimulant medications, cardiovascular disease, and no abnormal findings on the MRI [31]. Average age-group specific MRI template was nonlinearly registered to an average reference image using “Advanced Normalization Tools” (ANTS) [32]. This program provides symmetric normalization of the source volumes to the reference volumes. The data consisted of average (male and female) T1-weighted MRI for the head and brain, and segmenting priors for gray matter (GM), white matter (WM), and cerebrospinal fluid (CSF), from the Neurodevelopmental MRI Database [31,33,34,35,36]. The head model is shown in Figure A1 of Appendix B.

Tetrahedral volume mesh was created using the ROAST toolbox [27], which is a Matlab (Mathworks, MA, USA) script based on three open-source software; Statistical Parametric Mapping (SPM) [37], Iso2mesh [38], and getDP [39]. ROAST used SPM12 [40] to segment the head and the brain. After segmentation, five tissues were labeled for the tetrahedral volume mesh, namely, Scalp, Skull, Cerebrospinal Fluid (CSF), Gray Matter (GM), and White Matter (WM). These different brain tissues for the volume mesh were modeled as different volume conductors for finite element analysis (FEA) in the ROAST. Here, isotropic conductivity based on prior works was used for different brain tissues [30] which were (in S/m): Scalp = 0.465; Skull = 0.01; CSF = 1.654; GM = 0.276; WM = 0.126 [7,30,41,42]. A subject-specific cap fitted to the individual head model was created using the high-density 10-05 EEG locations [43], EGI net-based system (https://www.egi.com), and extra electrodes from ROAST [27] along with nine custom locations that were defined on the neck and the lower head.

### 2.3. Finite Element Analysis (FEA) for ctDCS Optimization Based on the MRI Template of 55 to 59 Years Old

Although multi (>2)-electrode montages can improve the focality and specificity [44] and can be delivered by our (expensive) stimulation device (STARSTIM 8, Neuroelectrics, Spain), however, we were limited by a 2-electrode bipolar montage so that the ctDCS montage can be translatable to low-cost (<$150) tDCS devices available in community setting (or, home-based) in India. Furthermore, whole head subject-specific MRI-based head modeling may not be feasible in a low-resource setting constrained by a lack of computing power so we aimed to identify a reduced set of electrodes optimized for ctDCS. Then, based on that reduced set of electrodes, we aimed to identify “one-size-fits-all” bipolar montage across our post-stroke subject group (*n* = 10) that can maximally (electric field strength) target bilateral dentate nuclei (postulated to affect motor as well as cognitive function [13]) or can uniformly (comparable electric field in X, Y, Z directions across lobules VII-IX) target the leg area of the cerebellum [7]. Therefore, the first step was to perform computational modeling across different available bipolar ctDCS montages and ctDCS optimization based on an age-appropriate averaged (*n* = 73) MRI template of the 55 to 59 years old. Here, tetrahedral volume meshing and the FEA was performed using the ROAST pipeline [27]. This pipeline provides a numerical tool to solve the required partial differential equations (PDE) to generate the transfer matrices necessary for the optimization [7]. Boundary condition was set to constant injection current (Neumann boundary condition). The electric field (EF) was modeled for ctDCS using five different montages.

#### 2.3.1. Computational Modeling and Optimization Based on MRI Template of 55 to 59 Years Age-Group

(1) Celnik montage [13]: 5 cm × 5 cm anode was placed over the right cerebellum, 1 cm below, and 3 cm lateral to the inion (Iz, 10/10 EEG system). The 5 cm × 5 cm cathode was over the right buccinator muscle for ctDCS with 2 mA direct current.

(2) Manto montage [45]: 5 cm × 5 cm anode was placed over the right cerebellum, 1 cm below, and 3 cm lateral to the inion (Iz, 10/10 EEG system). The 5 cm × 5 cm cathode was on the contralateral supraorbital area (FP2, 10/10 EEG system) for ctDCS with 2 mA direct current.

(3) Extracephalic montage: 5 cm × 5 cm anode was placed over the right cerebellum, 1 cm below, and 3 cm lateral to the inion (Iz, 10/10 EEG system). The 5 cm × 5 cm cathode was on the right neck area for ctDCS with 2 mA direct current.

(4) Optimization for dentate nuclei: electrode location of one 3.14 cm^2^ (1 cm radius) circular anode and one 3.14 cm^2^ circular cathode was optimized using our computational pipeline [7] for 2 mA ctDCS. The details on the optimization process are presented next in Section 2.3.2.

(5) Optimization for bilateral lobules VII-IX: electrode location of one 3.14 cm^2^ (1 cm radius) circular anode and one 3.14 cm^2^ circular cathode was optimized using our computational pipeline [7] for 2 mA ctDCS. The details on the optimization process are presented next in Section 2.3.2.

#### 2.3.2. Cerebellar tDCS Optimization Using the Head Model for the Age Group of 55–59 Years

Finite element analysis (FEA) tools, including ROAST [27], can be used to solve the quasistatic approximation for Maxwell’s equation with a linear approximation of Ohm’s law in a purely resistive medium Ω So, we can write in a matrix form $\vec{E}=LI$ where $\vec{E}$ is the electric field vector ($\vec{E}=\left[E_{x}\;E_{y}\;E_{z}\right]$); x, y, z are 3D global Cartesian coordinates—see Figure 2) generated by the stimulation currents, I, applied at the scalp electrode array, and L is the ‘transfer matrix’ (or, ‘leadfield matrix’) that (columns) maps the electric field ($\vec{E}=\left[E_{x}\;E_{y}\;E_{z}\right]$); generated in the brain for an unit current applied to each of the stimulation electrodes [46] with a joint return electrode (‘Cz’ in our case [7]). Here, the headspace is discretized as a 3D finite element mesh and the discretized solution for $\vec{E}$ can be availed after FEA at the nodes (called the nodal values). With discretization, the transfer matrix, L, is a 3*m* × *n* size matrix for *m* nodes and *n* scalp stimulation current sources (excluding ‘Cz’), I. Therefore, the forward model can be written as, $\vec{E}=LI+e,$ where the 3*m* × *n* electric field vector, $\vec{E}$, at any node, *m*, is a linear projection or mapping by the transfer matrix, L, of the stimulation current sources, I, with additive (environmental) noise, e. Here, usually *m >> n*, due to large (>10000) number of nodes, *m*, necessary to reduce numerical error in FEA [47] when compared to the number of electrode locations that available on the EEG cap (=429 in our case—See Appendix B: Table A2). In fact, the current applied at a finite-sized electrode (1cm circular electrode in our case) needs to be resolved to the nodes at the electrode-scalp interface, so one can also use uniformly distributed nodes at the scalp surface mesh for point current sources, I, to generate the ‘transfer matrix’ which can however drastically increase *n* and can make the system underdetermined requiring regularization as discussed next.

We can assume that the additive noise, e, is a multivariate Gaussian variable with zero mean and covariance matrix, $C_{e},$ which is independent of I. The additive noise can be due to external electromagnetic sources (can be recorded on the scalp during the experiment) in the absence of any applied scalp stimulation current sources, I. The problem of finding appropriate stimulation currents, $I\in R^{n}$, i.e., the vector of the unknown, for the multi-electrode array to shape the given nodal values of the electric field, $\vec{E}\in R^{3m}$, via the transfer matrix, $L\in R^{3m\times n}$, in presence of noise, $e\in R^{3m}$, can be framed as a minimization problem with L_2_ regularization [48], $\underset{I}{\hat{I}=\arg\;\min}\left({\left(\vec{E}-LI\right)}^{T}C_{e}{}^{-1}\left(\vec{E}-LI\right)+\lambda {\left\|I\right\|}_{2}^{2}\right)=L{\left(L^{T}L+\lambda C_{e}\right)}^{-1}\vec{E}$, where ( )^T^ is the transpose of the matrix, λ is the penalization parameter to keep the stimulation currents, $\hat{I}$, small, and $C_{e}$ is the 3*m* × *n* noise covariance matrix. Here, the solution, $\hat{I}$, emphasizes stimulation current sources near the peak (target) electric field, $\vec{E}$, which is driven by the norms of the columns of the transfer matrix, L. Here, L is usually very large and sparse as computed from FEA. Current sources for a superficial (near the scalp) brain target (peak electric field, $\vec{E}$) are desired to be near the peak electric field, $\vec{E}$, in practice (e.g., to stay away from non-targeted lesions), therefore, dropping a scalp stimulation current source with the high norm of its corresponding column inc L will be detrimental. So, a forward selection approach to find the scalp stimulation current sources with the high norm of its corresponding L column can be applied to reduce the number of variables, i.e., the size of the vector $I\in R^{n}$. We applied this approach to select the size of vector $I\in R^{n}$ that is appropriate for our cerebellar target (here, “superficiality” of the target is in terms of the resistivity of the medium where a similar target depth in a conductive medium will be more superficial than a resistive medium). We also assumed that the covariance matrix, $C_{e},$ is an identity matrix, so $\underset{I}{\hat{I}=\arg\;\min}\left({\left(\vec{E}-LI\right)}^{T}\left(\vec{E}-LI\right)+\lambda {\left\|I\right\|}_{2}^{2}\right)$ can be framed as a convex optimization with constraints [49] where $\hat{I}$ vector is the optimization variable. Here, convex optimization with constraints [49] is a powerful technique to minimize functions, *f*, that are convex, i.e., *f*(α*x* + β*y*) ≤ α*f*(*x*) + β*f*(*y*), for all $x,\;y\in R^{n}$ and all α, β∈R with α + β = 1, α ≥ 0, β ≥ 0. Convex optimization is not only applicable for least-square regression problem shown above but also for linear programming where the objective is to maximize the electric field, $\vec{E}$, at the targeted nodes of the brain region based on a vector of weights, $W\in R^{3m}$, i.e., $\underset{I}{\hat{I}=\arg\;\max}\left(W^{T}LI\right)$. The ‘beamforming’ problem in array signal processing [46,50] based on the minimization of the total energy stored in an electric field constrained to a target electric field, $\vec{E}$, at a volume is equivalent to the least-square problem (can be shown using Lagrange multipliers) [49].

In this study, we formulated two convex optimization problems [49] based on the head model for the age group of 55–59 years from the Neurodevelopmental MRI Database [31,33,34,35,36] (see Section 2.2 for details),

Objective 1: minimize the sum of squares of error between the desired electric field distribution, $\vec{E}$, at the ankle/leg area of the cerebellum (i.e., bilateral cerebellar lobules VII-IX [7]) and the one generated by the stimulation currents, i.e., $\underset{I}{\hat{I}=\arg\;\min}\left({\left(\vec{E}-LI\right)}^{T}\left(\vec{E}-LI\right)\right)\underset{I}{=\arg\;\min}{\left\|\vec{E}-LI\right\|}^{2}$, under the following constraints:

Total anodal current is equal to the cathodal current;

$$\overset{n}{\underset{j=1}{\sum }}I_{i}=0$$

Total anodal and cathodal current magnitude is below a set threshold of 4 mA for safety and comfort (i.e., maximum anodal or cathodal current is 2 mA);

$$\overset{n}{\underset{j=1}{\sum }}\left|x_{j}\right|\leq 4$$

This convex optimization problem was solved using CVX toolbox in MATLAB (Mathworks, Inc., MA, USA) to get an achievable uniform ($E_{x}=E_{y}=E_{z}$) electric field at the cerebellar lobules related to the ankle/leg function [51], a.k.a, lobules VII-IX [7], that can then be scaled in practice by scaling the stimulation currents, $\hat{I}$, vector due to a linear system, $\vec{E}=LI$.

Objective 2: maximize the electric field, $\vec{E}$, at the dentate nuclei of the cerebellum, i.e., $\underset{I}{\hat{I}=\arg\;\max}\left(W^{T}LI\right)$ where $W\in R^{3m}$ is a vector of weights (with one for $E_{x},E_{y},E_{z}$ at the nodes of the dentate nuclei and zeros elsewhere), under the following constraints:

Total anodal current is equal to the cathodal current;

$$\overset{n}{\underset{i=1}{\sum }}I_{i}=0$$

Total anodal and cathodal current magnitude is below a set threshold of 4 mA for safety and comfort (i.e., maximum anodal or cathodal current is 2 mA);

$$\overset{n}{\underset{j=1}{\sum }}\left|x_{j}\right|\leq 4$$

This was solved using CVX toolbox in MATLAB (Mathworks, Inc., MA, USA) to get a maximum electric field at the dentate nuclei of the cerebellum, that can then be scaled in practice by scaling the stimulation currents, $\hat{I}$, vector due to a linear system, $\vec{E}=LI$.

Possible electrode positions for stimulation current sources were defined for the whole head coverage (*n* = 429—see Appendix B: Table A2) by combining the high-density 10-05 EEG locations [43] with the EGI net-based system (https://www.egi.com) and extra electrodes from ROAST [27]. CLOS pipeline [7] was used to compute the ‘transfer matrix’, L, for each nodal location and direction of the electric field by combining 429 FEA simulations. In all the simulations, the voxel size was considered as 1mm^3^. In CLOS [7], the electric field, $\vec{E}$, a vector can be mapped (i.e., $A\vec{E}=ALI$) using a spatially unbiased atlas template of the cerebellum and brainstem (SUIT) [52] for 34 SUIT parcellations (or, regions—see Appendix A: Table A1) and the non-cerebellar brain (i.e., total 35 regions) to the average electric field in the X, Y, and Z directions of the global coordinate system, i.e., mean Ex, mean Ey, mean Ez, in the 35 regions (where A is 105 × 3*m* mapping matrix). We divided the new transfer matrix, AL (reduced size and sparsity from L but easier to process in Matlab with limited desktop memory) into three 35 × 429 matrices, each for mean Ex, mean Ey, and mean Ez, as shown in Appendix C.

#### 2.3.3. Computational Modeling for the Post-Stroke Subjects

**Figure 3:**
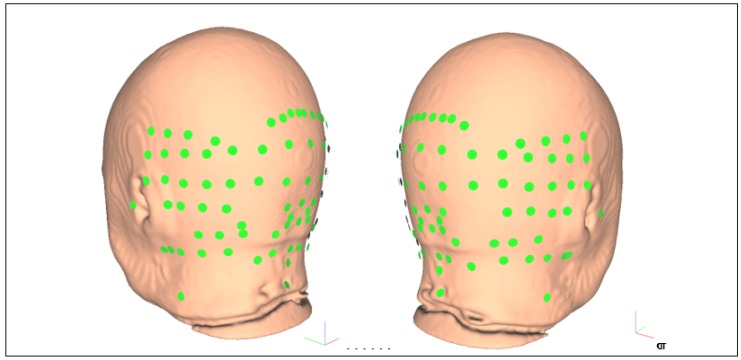
Reduced set of 87 electrode locations to optimize bipolar ctDCS montage for stroke survivors, namely: “E145”, “E146”, “E156”, “E165”, “Ex1”, “Ex2”, “Ex3”, “Ex4”, “Ex5”, “Ex6”, “Ex7”, “Ex8”, “Exx10”, “Exx11”, “Exx12”, “Exx1”, “Exx2”, “Exx3”, “Exx4”, “Exx5”, “Exx6”, “Exx7”, “Exx8”, “Exx9”, “Exxz”, “Exz”, “I1h”, “I2h”, “Iz”, “NkB”, “NkL”, “NkR”, “O1”, “O1h”, “O2”, “O2h”, “OI1”, “OI1h”, “OI2”, “OI2h”, “OIz”, “Oz”, “P10”, “P10h”, “P7”, “P7h”, “P8”, “P8h”, “P9”, “P9h”, “PO10”, “PO10h”, “PO7”, “PO7h”, “PO8”, “PO8h”, "PO9”, “PO9h”, “POO10”, “POO10h”, “POO1h”, “POO2”, “POO2h”, “POO3h”, “POO8”, “POO9”, “POO9h”, “POOz”, “PPO10”, “PPO10h”, “PPO7”, “PPO7h”, “PPO8”, “PPO8h”, “PPO9”, “PPO9h”, “T5”, “T6”, “TPP10h”, “TPP7”, “TPP8”, “TPP8h”, “TPP9h”, “Z1”, “Z2”, “Z7”, “Z9”. The global coordinate system shown by colored vectors, red: X, green: Y, blue: Z.

**Figure A3:**
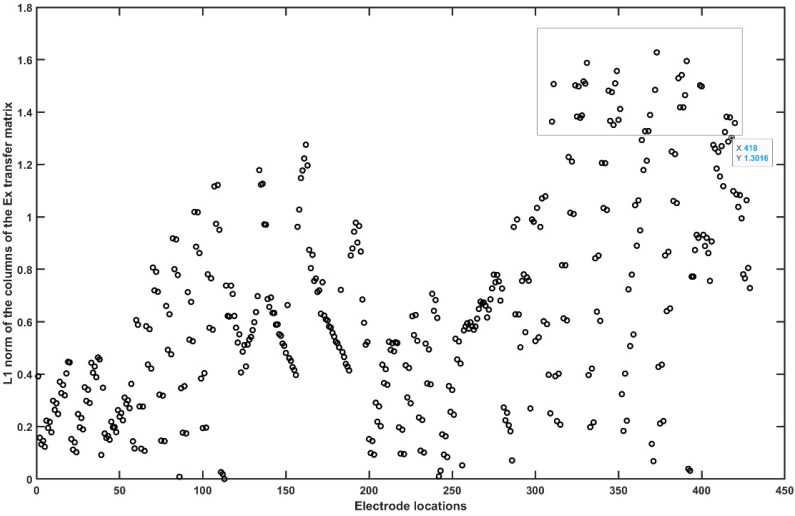
L1 norm of the columns of the Ex transfer matrix without the non-cerebellar brain (i.e. row 35). The rectangle shows the electrode locations with the high L1 norm (electrodes relevant for cerebellar stimulation in Ex).

**Figure A4:**
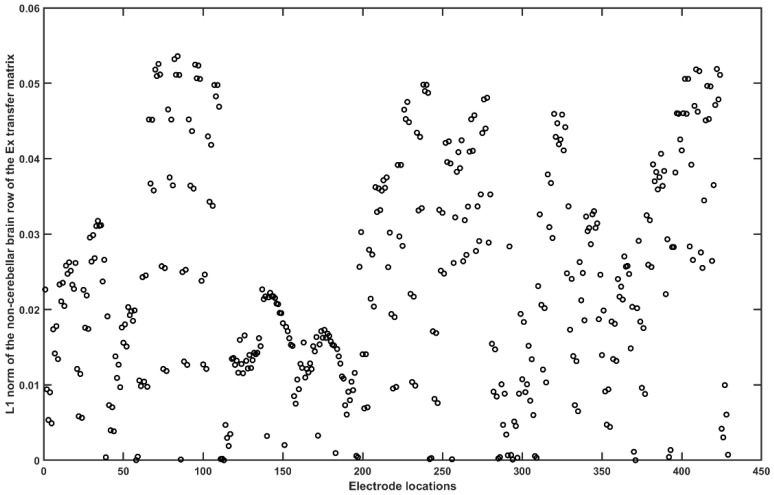
L1 norm of the non-cerebellar brain row (= 35) of the Ex transfer matrix.

**Figure A6:**
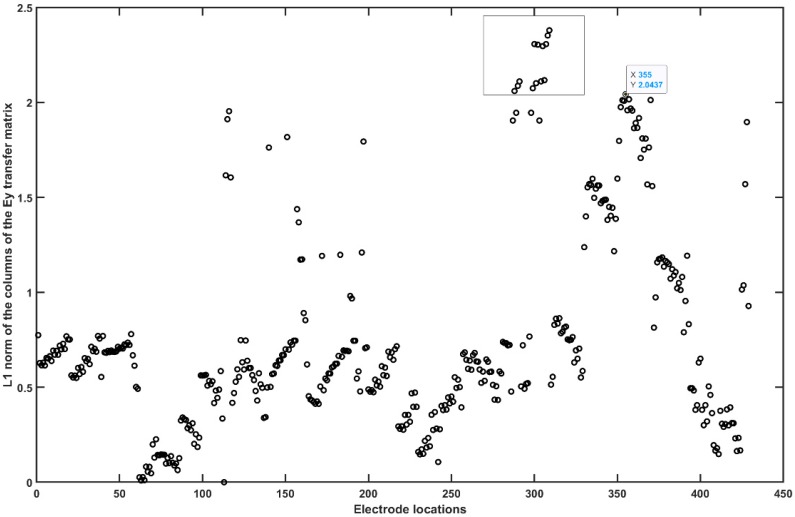
L1 norm of the columns of the Ey transfer matrix without the non-cerebellar brain (i.e. row 35). The rectangle shows the electrode locations with the high L1 norm (electrodes relevant for cerebellar stimulation in Ey).

**Figure A7:**
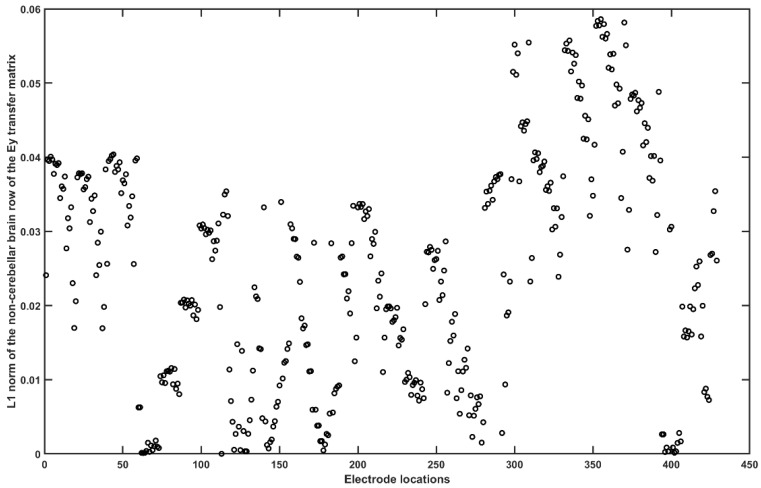
L1 norm of the non-cerebellar brain row (= 35) of the Ey transfer matrix.

**Figure A9:**
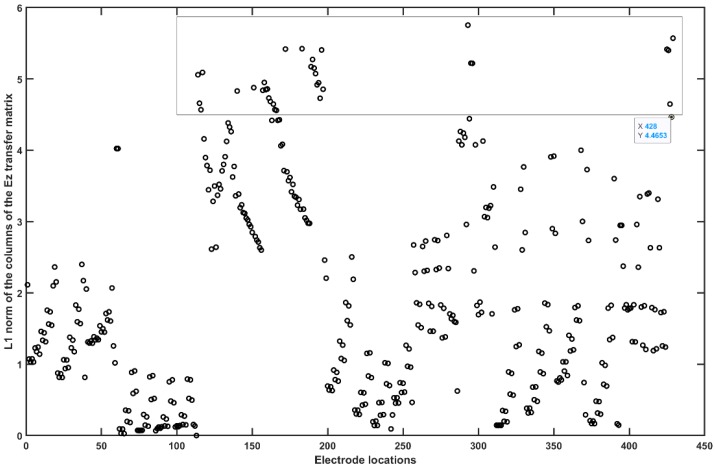
L1 norm of the columns of the Ez transfer matrix without the non-cerebellar brain (i.e., row 35). The rectangle shows the electrode locations with the high L1 norm (electrodes relevant for cerebellar stimulation in Ez).

**Figure A10:**
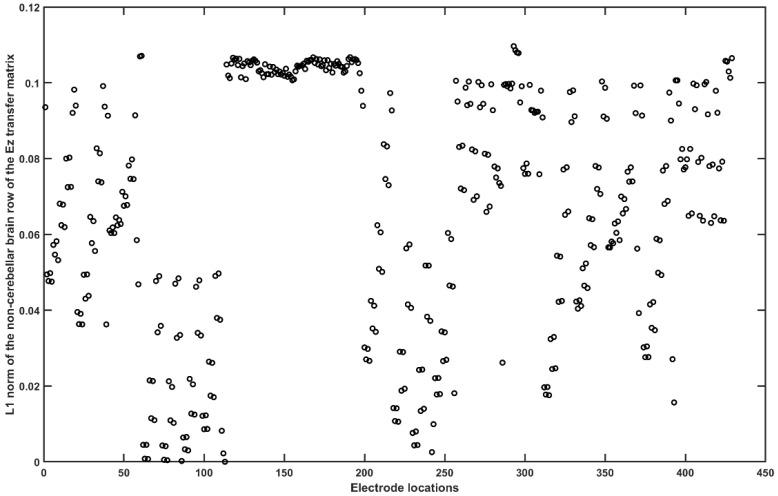
L1 norm of the non-cerebellar brain row (= 35) of the Ez transfer matrix.

Stroke survivors had lesions in the cerebral areas (primarily frontal lobe) so the bipolar electrodes needed to be limited to the scalp overlying the cerebellum and the neck. The L1 norm of the columns of these transfer matrices (from 55–59 years age-group MRI template) are shown in Figure A3, Figure A6 and Figure A9 for Ex, Ey, and Ez respectively in Appendix B along with a reduced set of electrodes (see Figure 3) found from the union of the electrode locations with high L1 norm from the three (Ex, Ey, and Ez) transfer matrices (see Table A3, Table A4 and Table A5 for Ex, Ey, and Ez respectively in Appendix C). Here, L1 norm of the non−cerebellar brain (row= 35) of the Ex, Ey, and Ez transfer matrices are lower than 0.12 compared to greater than 1 for the cerebellar brain (see Figure A4, Figure A7 and Figure A10 for Ex, Ey, and Ez respectively in Appendix C) so not affected significantly by the reduced set of electrodes (see Figure 3).

#### 2.3.4. Assessing the Electric Field Distribution in the Cerebellar Lobules

FEA was performed with post-stroke MRIs (*n* = 10) using this reduced set of electrodes in the low-resource (point-of-care) setting constrained by a lack of computing power to generate the transfer matrices from the subject-specific post-stroke head models. Following FEA in ROAST, we used SUIT [52,53] to normalize the cerebellar electric field distribution. T1-weighted images were fitted to the SUIT template of the human cerebellum in SPM12 [40]. The cerebellar mask was visually checked in MRIcron (http://www.diedrichsenlab.org/imaging/propatlas.htm). Non-linear deformation was then applied to each electric field image obtained from ROAST. The volume of the cerebellar lobules, defined by the SUIT atlas [52], was used for the extraction of the lobular electric field distribution. We customized SUIT codes to assess the electric field distribution in the two dentate nuclei in addition to the 28 lobules. Here, vermis areas were excluded from further analysis. This reduced set of electrodes (see Figure 3) was faster to process using CVX (toolbox in MATLAB, Mathworks, MA, USA) with affine constraints [49] for ctDCS optimization. The resultant group-averaged bipolar montage (shown in Figure 2) was used on the 5 post-stroke participants who volunteered for ctDCS and FRT study (starred subjects in Table 1). So, we applied two bipolar montages limited to scalp overlying the cerebellum and the neck as listed below.

(1) Bipolar PO9h–PO10h montage for dentate nuclei: A 3.14 cm^2^ (1 cm radius) circular anode was placed at the contra-lesional side, and a 3.14 cm^2^ cathode was placed at the ipsilesional side for ctDCS with 2 mA direct current.

(2) Bipolar Exx7–Exx8 montage for bilateral leg lobules VII-IX: A 3.14 cm^2^ (1 cm radius) circular anode was placed at the contra-lesional side, and a 3.14 cm^2^ cathode was placed at the ipsilesional side for ctDCS with 2 mA direct current.

The bipolar electrode montage was modeled in ROAST at the given scalp locations to compute the electric field in the brain tissues [54]. In all simulations, the voxel size was considered as 1 mm^3^.

#### 2.3.5. Assessing the Electric Field Distribution in the Occipital and Parietal Lobes

It was important to assess the electric field distribution in the cerebral volumes near the cerebellum for safety (avoid spillover to the lesioned brain). To evaluate the electric field distribution of the nearby occipital and parietal lobes, we created a mask for each lobe using MNI atlas in the FSL [55]. A code was scripted in MATLAB to isolate the electric field in the masked regions (occipital and parietal lobes) of the brain.

### 2.4. Statistical Analysis of the Electric Field Distribution in the Head Model of the 55 to 59 Years Old

The electric field was computed at all the voxels (voxel size 1 mm^3^) using ROAST [27] for the five montages (see Section 2.3.1). We analyzed the electric field distribution across lobules, dentate nuclei, and occipital and parietal lobes using two-way ANOVA (‘anovan′ in MATLAB) for the factors of interest – brain regions, montages, and their interactions (brain region*montage). In the Generalized Linear Model (GLM), the proportion of the total variability in the dependent variable that is accounted for by the variation in the independent variable found using the eta-squared effect size measure. Post-hoc multiple comparison tests were conducted using Bonferroni′s critical values.

### 2.5. Regression Analysis of the Electric Field Distribution with the Behavioral Outcome in the Post-Stroke Subjects

Recent work shows that the cerebellum is organized in distinct functional subregions revealed by a multi-domain task battery (MDTB) based on Diedrichsen and Kriegeskorte [56,57] that provided a functional atlas. Therefore, ctDCS can have a multi-domain functional effect that can be elucidated with a multi-domain task battery (MDTB) [57] where a functional atlas can be used for the first optimization of ctDCS electrode montage. Here, the novel parcellation of the human cerebellum into functional regions using MTDB can be scaled as the spatial target for the electric field, $\vec{E}$, where $\vec{E}$ vector can be constrained to be normal to the cerebellar surface in order to optimally target the Purkinje cells [7]. MDTB results also revealed a need for representational models that specify how the electric field, $\vec{E}$, distribution due to ctDCS relates to motor responses or cognitive processes across MDTB, i.e., the distribution of activity profiles across experimental conditions. In the current study, we hypothesized that the electric field, $\vec{E}$, distribution due to ctDCS has a linear relationship with the *q* behavioral outcome measures across *p* subjects (or, *p* trials of a single subject) represented by *p × q* behavioral outcome (continuous) matrix, B. Linear model, when applicable, needs to be appropriately regularized, which effectively imposes a prior on the activity profiles. Such a linear relationship can be captured by *p × q ×* 3*m* regression matrix, A, i.e., $B=A\vec{E}+w$, where w is the zero-mean normally distributed residuals not explained by the linear regression. Here, *p × q* behavioral outcome (continuous) matrix, B, can suffer from multicollinearity in a large multi-domain task battery so we may be able to reduce its dimension using principal component analysis and then the orthogonal dependent variables (i.e., uncorrelated functional profiles) can be individually fitted to the electric field, $\vec{E}$, distribution as a predictor.

In our repeated-measure counter-balanced crossover study, we compared two bipolar montages across 5 post-stroke subjects (“one-size-fits-all”), bipolar PO9h–PO10h for dentate nuclei and bipolar Exx7–Exx8 for bilateral cerebellar leg lobules VII–IX, so the proportion of the total variability in the electric field, $\vec{E}$, is postulated to be accounted for by the variation in the independent variables, brain regions, montages, stroke participants, and their interactions. This leads to a GLM using three-way ANOVA (‘anovan’ in MATLAB) for the factors of interest – brain regions, montages, stroke participants, and their interactions – based on their statistical significance. Here, the electric field, $\vec{E}$, distribution is the dependent variable and different electric field, $\vec{E}$, distribution can affect outcomes across MTDB [57]. Next, the outcomes, B, can be treated as a random variable and the goal is to predict, for each possible outcome, the probability of an electric field, $\vec{E}$, distribution (or, related montages, I, if statistically significant from ANOVA) exhibiting that outcome. In the current study, we have one behavioral outcome, i.e., the number of successful target reaches (binomial distribution) during FRT trials, so *q* = 1. Here, the *p* × 1 behavioral outcome matrix, B, is the success rate at FRT trials post-intervention for the different electric field, $\vec{E}$, distribution. This leads to a GLM for the dependent variable, B, using probit link (‘glmfit’ in MATLAB) for the predictor of interest (z-value of a normal distribution) – electric field, $\vec{E}$, distribution. Baseline equivalence between the two groups was confirmed by the Wilcoxon rank-sum test (‘ranksum’ in Matlab).

We postulate that ctDCS optimization should be based on deficits in functional outcomes during quantitative multi-task evaluation due to cerebellar multiple functionalities [58] where ctDCS optimization can be based on mapping to universal cerebellar computations [58], e.g., relate to the executive cluster of the CCAS [59] in IMA. Importantly, King et al. recently showed that lobular boundaries commonly used to summarize functional data do not coincide with functional subdivisions [57]. Here, human dentate nuclei have also been found to be divided into three functional territories; default-mode, salience-motor, and visual brain networks [60]. Therefore, we propose a functional optimization of ctDCS for future clinical studies to maximize behavioral outcomes based on representational models. Here, the behavioral outcome (continuous) matrix, B, from a multi-domain task battery can be subjected to dimension reduction along the principal gradient, *P*, that can reveal mapping to universal cerebellar computations across multiple task domains. This may be related to cerebellar double motor representation (lobules I-VI and VIII), and its relationship with triple non-motor representation (lobules VI/Crus I, Crus II/VIIB, IX/X) where functional differences and similarities across these different representations were shown recently [61].

If the montages have a significant effect on, $\vec{E}$, and the linear model holds, i.e., $P=A\vec{E}+w$, then for the *p* × 1 principal gradient, *P*, the *p* × 3*m* regression matrix, $\underset{A}{\hat{A}=\arg\;\min}\left({\left(P-A\vec{E}\right)}^{T}C_{w}{}^{-1}\left(P-A\vec{E}\right)\right)$, for a residual covariance matrix, C_w_. Since, $\vec{E}=LI+e$, so P=ALI+(Ae+w), where the covariance of (Ae+w) is $C_{w}+{\mathrm{AC}}_{e}A^{T}$. Therefore, if $\hat{A}$ is known from regression analysis (e.g., Figure 8 for our single outcome measure in this study) then the L_2_ regularized [48] optimal stimulation current sources for a given principal gradient, *P*, (e.g., cognitive or motor [62] as necessary for rehabilitation) can be found as $\underset{I}{\hat{I}=\arg\;\min}\left({\left(P-\hat{A}\mathrm{LI}\right)}^{T}{\left(C_{w}+{\hat{A}C}_{e}{\hat{A}}^{T}\right)}^{-1}\left(P-\hat{A}\mathrm{LI}\right)+{\lambda \|I\|}_{2}^{2}\right)$ subjected to other constraints (see Section 2.3.2) under convex optimization [49]. Also, linear programming can maximize the *p* × 1 principal gradient, *P*, i.e., $\underset{I}{\hat{I}=\arg\;\max}\left(W^{T}\hat{A}\mathrm{LI}\right),$ where $W\in R^{p}$ is a vector of weights (unit vector in our case), under the following constraints used in this study:

Total anodal current is equal to the cathodal current;

$$\overset{n}{\underset{i=1}{\sum }}I_{i}=0$$

Total anodal and cathodal current magnitude is below a set threshold of 4 mA for safety and comfort (i.e., maximum anodal or cathodal current is 2 mA);

$$\overset{n}{\underset{j=1}{\sum }}\left|x_{j}\right|\leq 4$$

This can be solved using CVX toolbox in Matlab (Mathworks, Inc., MA, USA) for the stroke subjects based on a reduced transfer matrix, $\overset{´}{L}\in R^{3m\times r}$, where $I\in R^{r}$ and r<n, i.e., $\underset{I}{\hat{I}=\arg\;\max}\left(W^{T}\hat{A}\overset{´}{L}I\right)$, after the forward selection approach to find *r* < *n* scalp stimulation current sources with the high norm of its corresponding L column (from the head model for the age group of 55–59 years).

## 3. Results

Figure 2 shows the head model from the MRI template of 55-59 years age-group which was used to generate the whole head transfer matrices (429 electrodes—See Appendix B: Table A2) to optimize the bipolar ctDCS montage. The reduced set of 87 electrode locations to optimize bipolar ctDCS montage were selected for high L1 norm of the columns of the transfer matrices related to cerebellar brain, namely (shown in Figure 3), “E145”, “E146”, “E156”, “E165”, “Ex1”, “Ex2”, “Ex3”, “Ex4”, “Ex5”, “Ex6”, “Ex7”, “Ex8”, “Exx10”, “Exx11”, “Exx12”, “Exx1”, “Exx2”, “Exx3”, “Exx4”, “Exx5”, “Exx6”, “Exx7”, “Exx8”, “Exx9”, “Exxz”, “Exz”, “I1h”, “I2h”, “Iz”, “NkB”, “NkL”, “NkR”, “O1”, “O1h”, “O2”, “O2h”, “OI1”, “OI1h”, “OI2”, “OI2h”, “OIz”, “Oz”, “P10”, “P10h”, “P7”, “P7h”, “P8”, “P8h”, “P9”, “P9h”, “PO10”, “PO10h”, “PO7”, “PO7h”, “PO8”, “PO8h”, “PO9”, “PO9h”, “POO10”, “POO10h”, “POO1h”, “POO2”, “POO2h”, “POO3h”, “POO8”, “POO9”, “POO9h”, “POOz”, “PPO10”, “PPO10h”, “PPO7”, “PPO7h”, “PPO8”, “PPO8h”, “PPO9”, “PPO9h”, “T5”, “T6”, “TPP10h”, “TPP7”, “TPP8”, “TPP8h”, “TPP9h”, “Z1”, “Z2”, “Z7”, “Z9”. In this study, we also wanted a low L1 norm of the columns of the transfer matrices (L1 norm < 0.01 selected—see Appendix C) for the non-cerebellar brain (i.e., row 35—see Appendix C) to avoid spillover to lesional cerebral areas in stroke subjects which limited the available electrode locations to “E145”, “E146”, “E156”, “E165”,”Ex1”, “Ex2”, “Ex3”, “Ex4”, “Ex6”, “Exx1”, “Exx2”, “Exx3”, “Exx4”, “Exx5”, “Exx6”, “Exx7”, “Exx8”, “Exxz”, “Exz”, “I1h”, “I2h”, “Iz”, “NkB”, “NkL”, “NkR”, “O2h”, “OI1h”, “OI2h”, “OIz”, “Oz”, “POO1h”, “POO2”, “POO2h”, “POOz”, “Z1”, “Z2”, “Z7”, “Z9”. This provided a reduced set of scalp electrode locations (primarily overlying the cerebellum) for stroke subjects.

**Figure 4:**
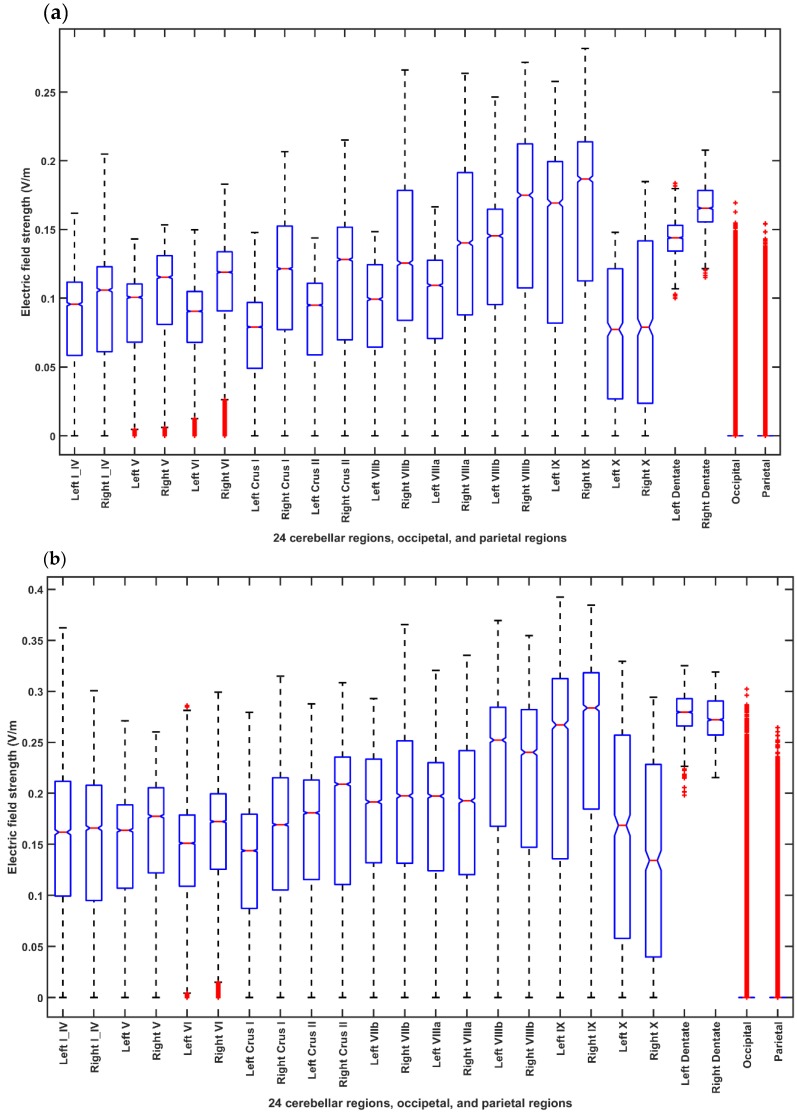

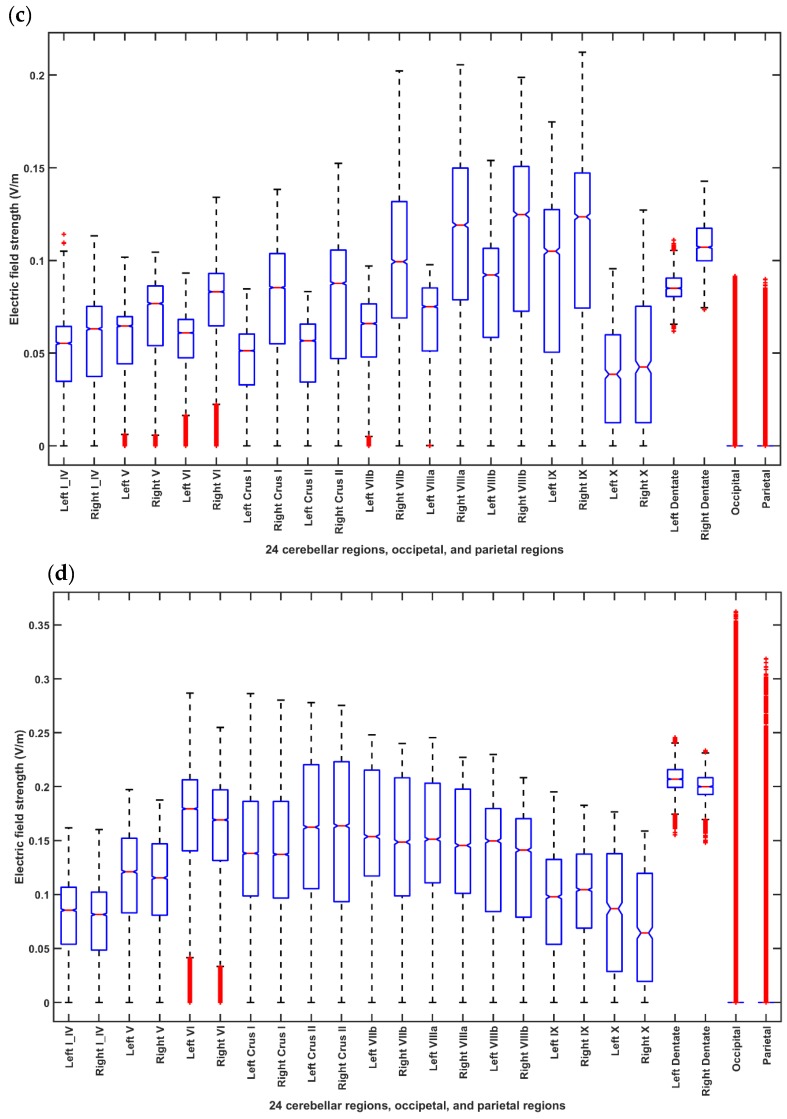

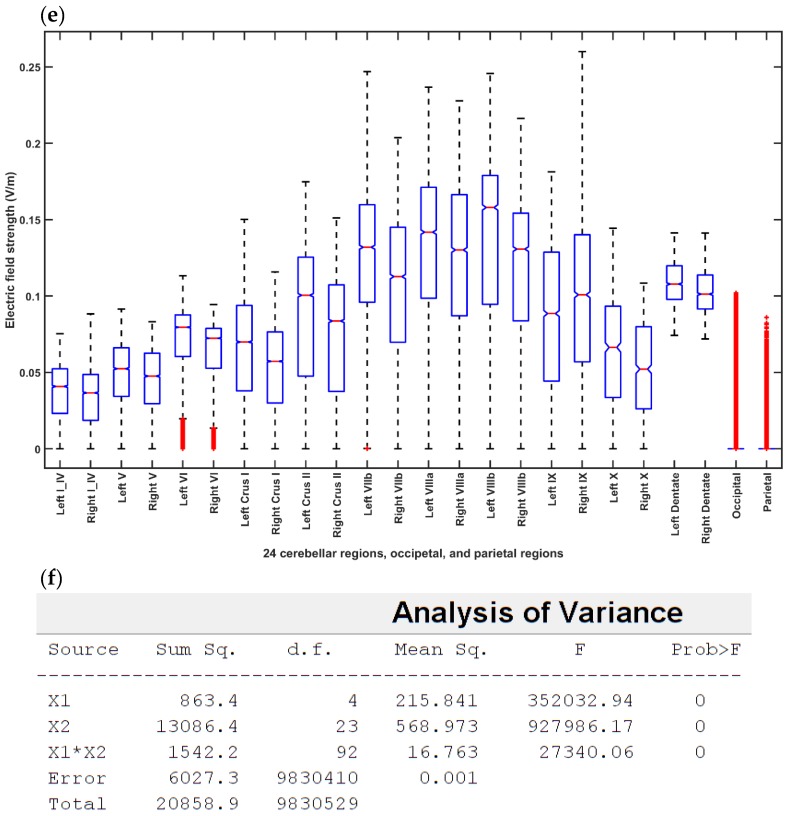
Boxplot of the electric field distribution for different ctDCS montages for the head model from the MRI template of 55-59 years age-group across 24 cerebellar regions, occipital and parietal lobes where in each box, the central mark indicates the median, and the bottom and top edges of the box indicate the 25th and 75th percentiles, respectively. The whiskers extend to the most extreme data points not considered outliers, and the outliers are plotted individually using the ‘+’ symbol. If the notches in the box plot do not overlap, one can conclude, with 95% confidence that the true medians do differ. (**a**) Shows the EF distribution for the Celnik montage. (**b**) Shows the EF distribution for the Manto montage. (**c**) Shows the EF distribution for the Extracephalic montage. (**d**) Shows the EF distribution for the PO9h–PO10h montage for case 1 (Optimization for dentate nucleus). (**e**) Shows the EF distribution for the Exx7–Exx8 montage for case 2 (Optimization for lobules VII–IX). (**f**) ANOVA table: Two-way ANOVA for the factors of interest–montages (X1), brain regions (X2), and their interactions (montage* brain region)–all found significant. Source: Source of variability; Sum Sq.: Sum of Squares due to each source; d. f.: Degrees of freedom associated with each source; Mean Sq.: Mean Square for each source, which is the ratio Sum Sq. /d. f. F: statistics which is the ratio of the mean squares.

The optimal bipolar montage found for the head model from the MRI template of 55-59 years age- group were Z7–POO2 for case 4 (optimization for dentate nuclei) and Exx5–Ex6 for case 5 (optimization for bilateral lobules VII–IX). For the post-stroke subjects undergoing ctDCS, we selected PO9h–PO10h for case 1 and Exx7–Exx8 for case 2 based on group-analysis of the subject-specific optimization since the lesional brain areas were primarily in the frontal lobe (occipital and parietal lobes were free from lesions) in the five post-stroke participants who volunteered for the ctDCS FRT study. Figure 4 shows the boxplot of the electric field (EF) strength for different ctDCS montages for the head model from the MRI template of 55-59 years age-group across 24 cerebellar regions, occipital and parietal lobes where Figure 4a shows the EF distribution for the Celnik montage, Figure 4b shows the EF distribution for the Manto montage, Figure 4c shows the EF distribution for the Extracephalic montage, Figure 4d shows the EF distribution for the PO9h–PO10h montage for case 1 (optimization for dentate nuclei), Figure 4e shows the EF distribution for the Exx7–Exx8 montage for case 2 (optimization for bilateral lobules VII–IX). Here, the electric field strength at the dentate nuclei was found to be high across all montages including the conventional Celnik and Manto ctDCS montages where the Manto montage from conventional ctDCS montages was found to be the best to stimulate the dentate nuclei in addition to the lower limb representations in the cerebellum. Also, the EF strength at the non-cerebellar occipital and parietal regions was found to be high (comparable to the Manto montage) for our PO9h–PO10h montage. However, the EF strength at the non-cerebellar occipital and parietal regions was found to be low (comparable to the Extracephalic montage) for the Exx7–Exx8 montage. Nevertheless, the median of the EF strength at the non-cerebellar occipital and parietal regions was low (<0.02 V/m), and most of the boxplot consisted of the outliers which are plotted individually using the + symbol. Here, Figure 4f shows two-way ANOVA results for the factors of interest–brain regions, montages, and their interactions (brain region*montage) which were all significant.

**Figure 5:**
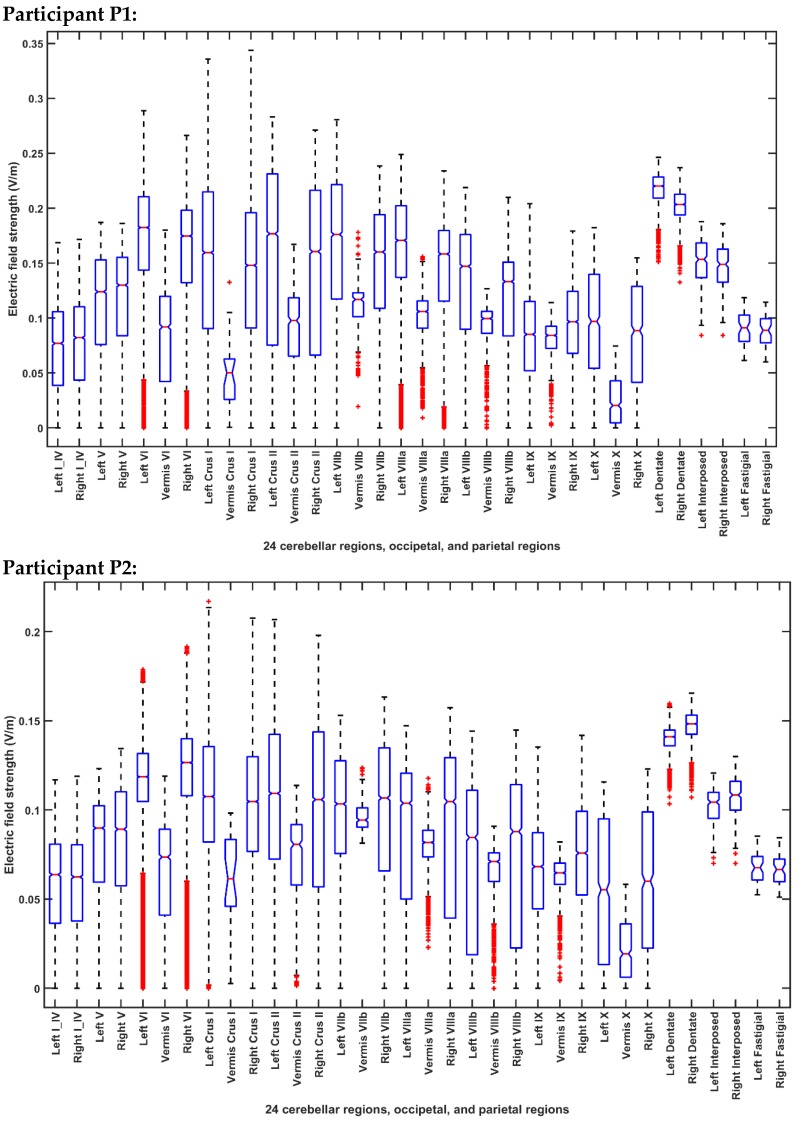

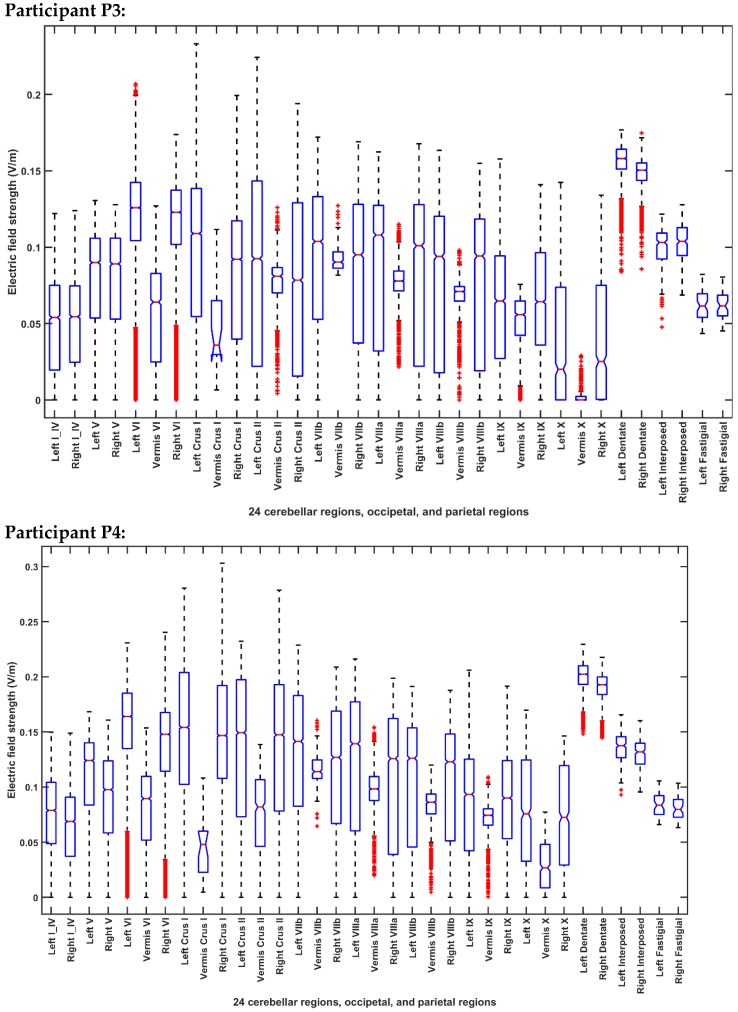

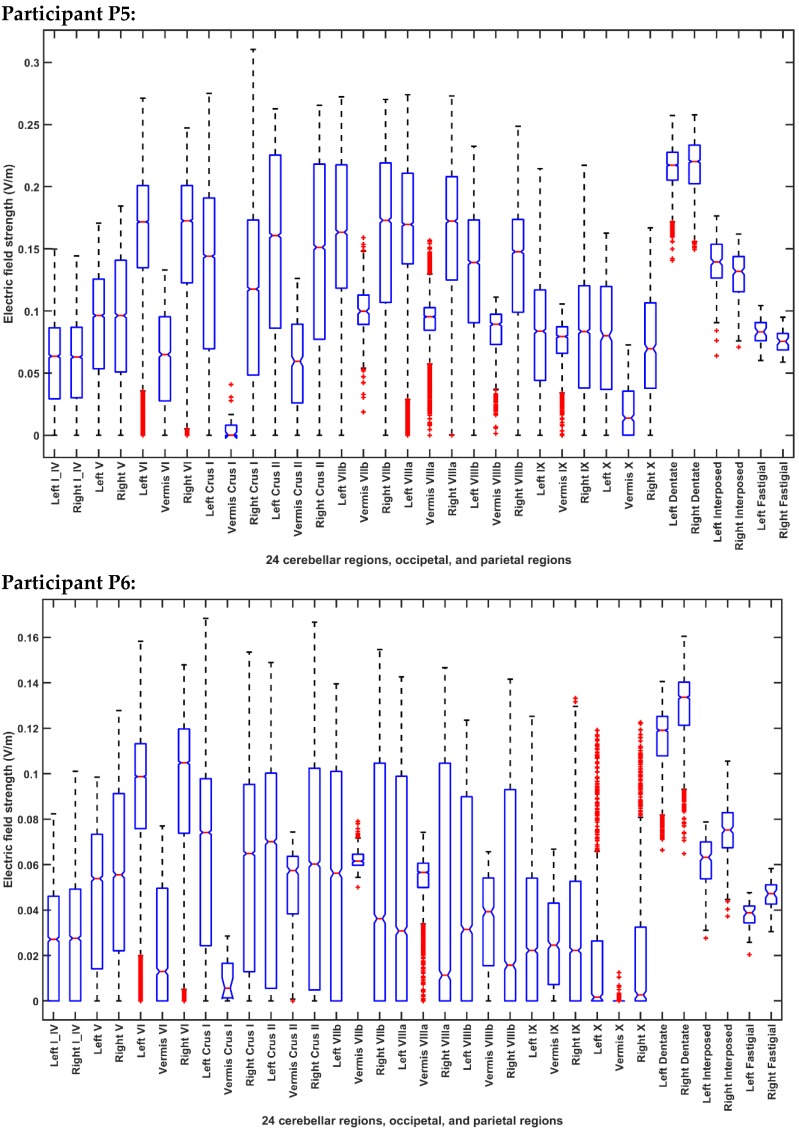

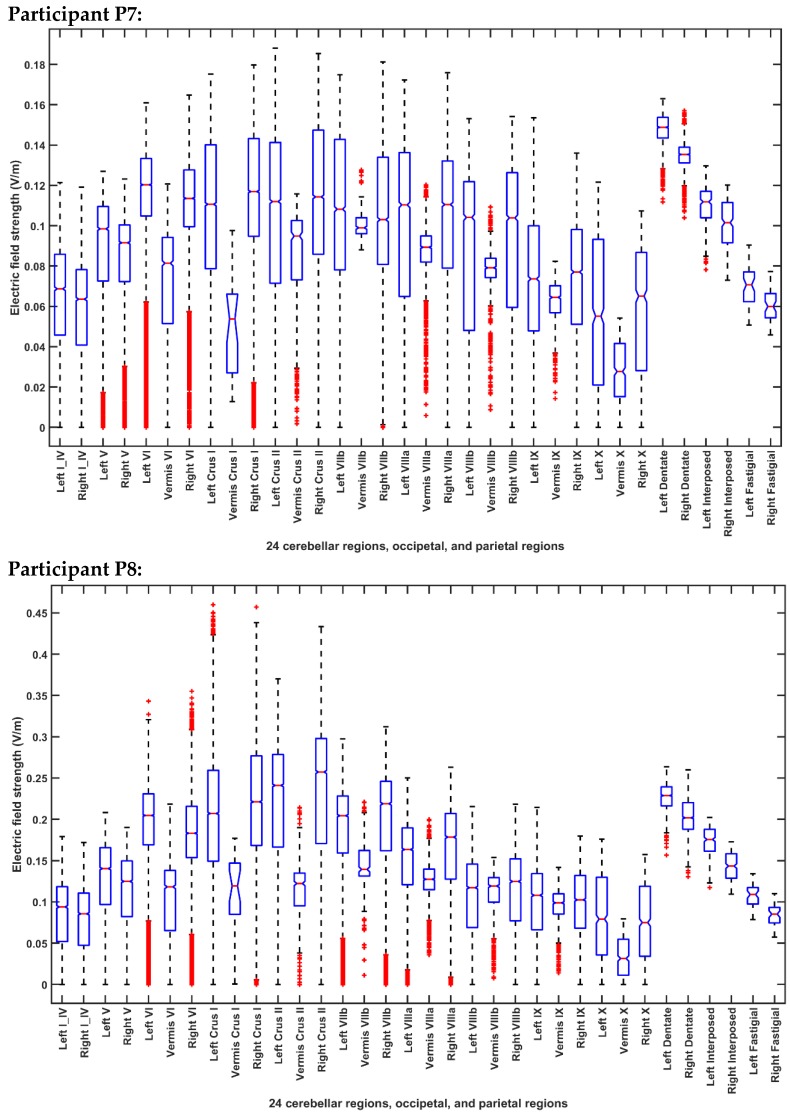

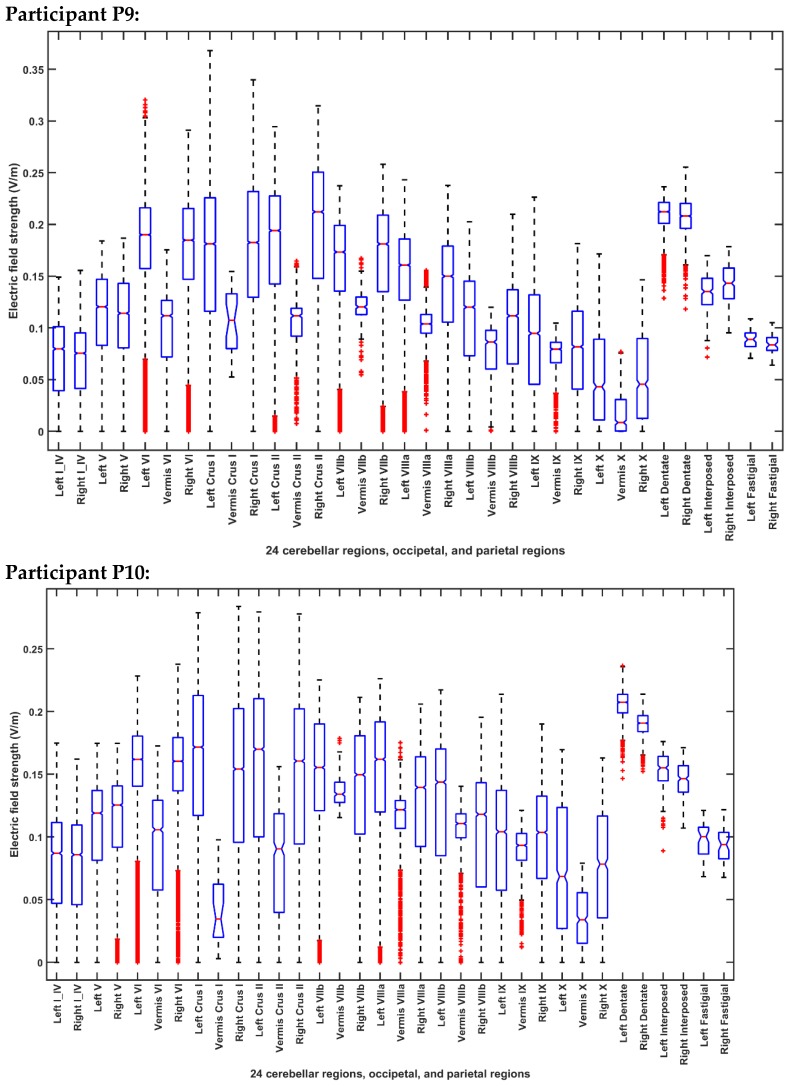
Boxplot of the lobular electric field distribution for the 10 post-stroke patients for the PO9h–PO10h montage for case 1 (optimization for dentate nuclei).

**Figure 6:**
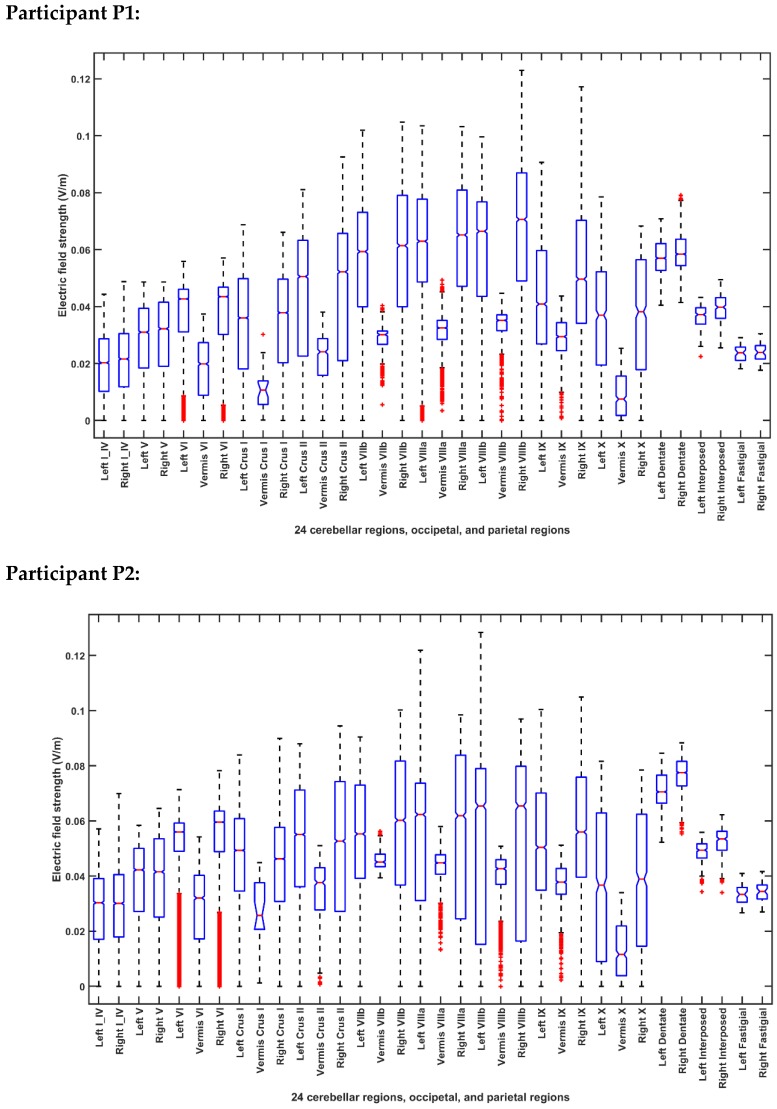

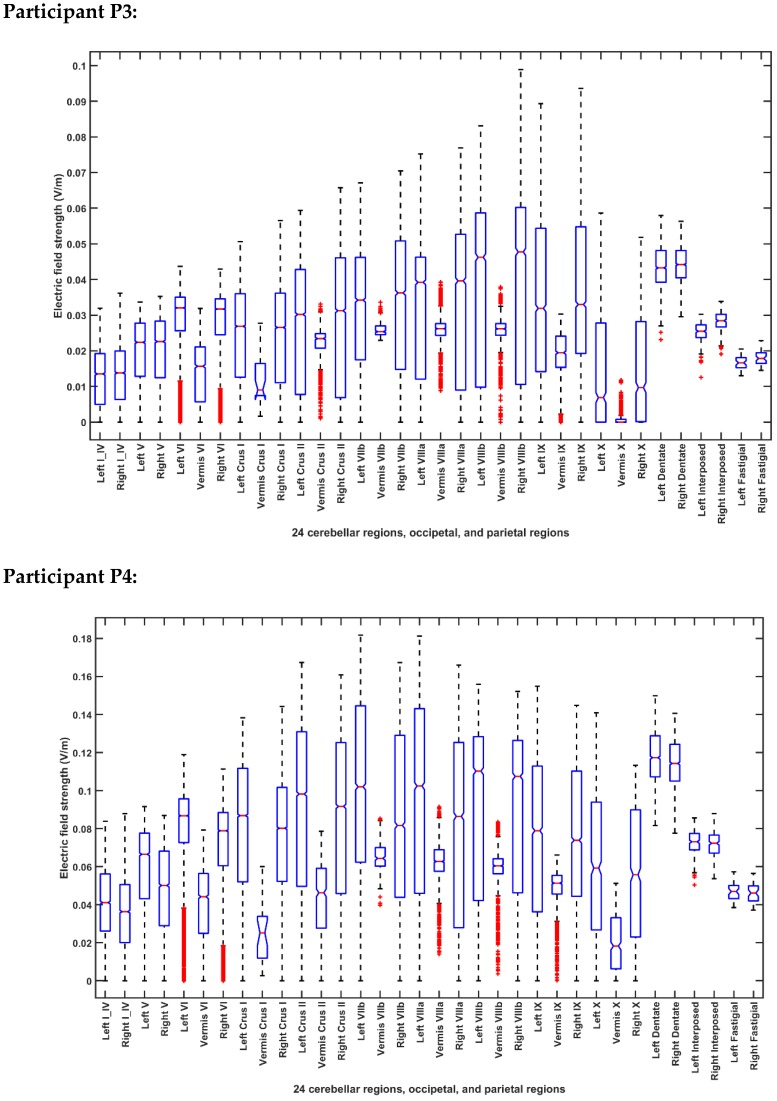

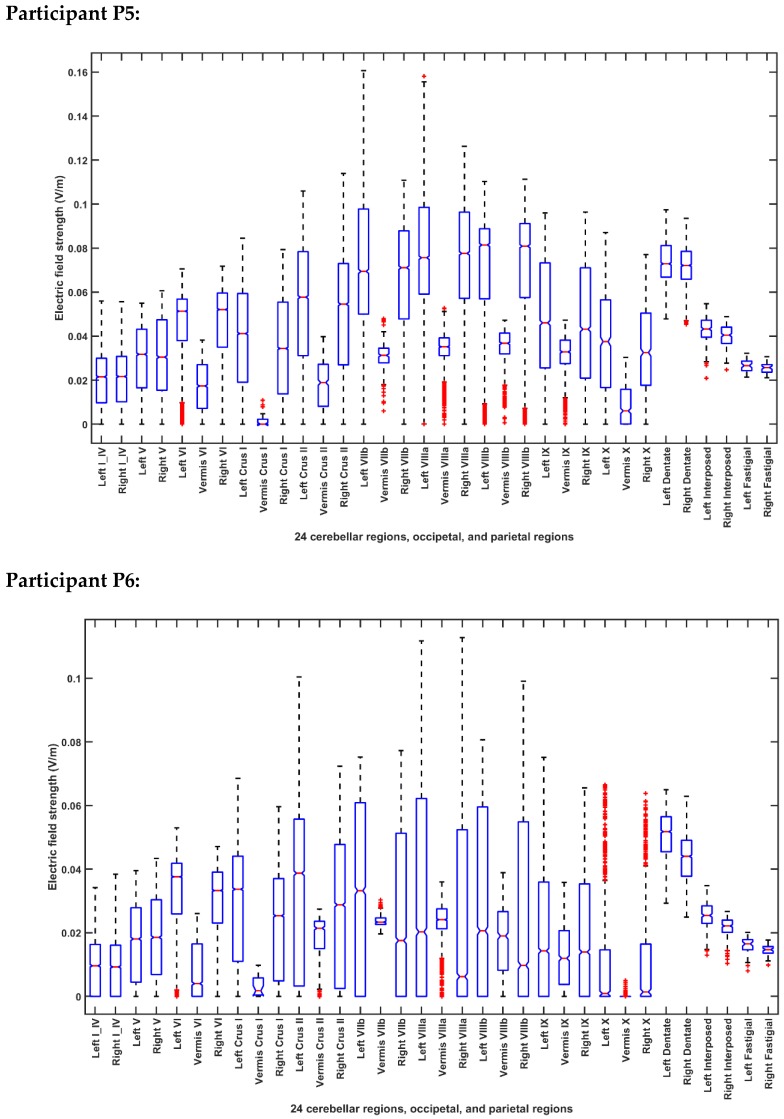

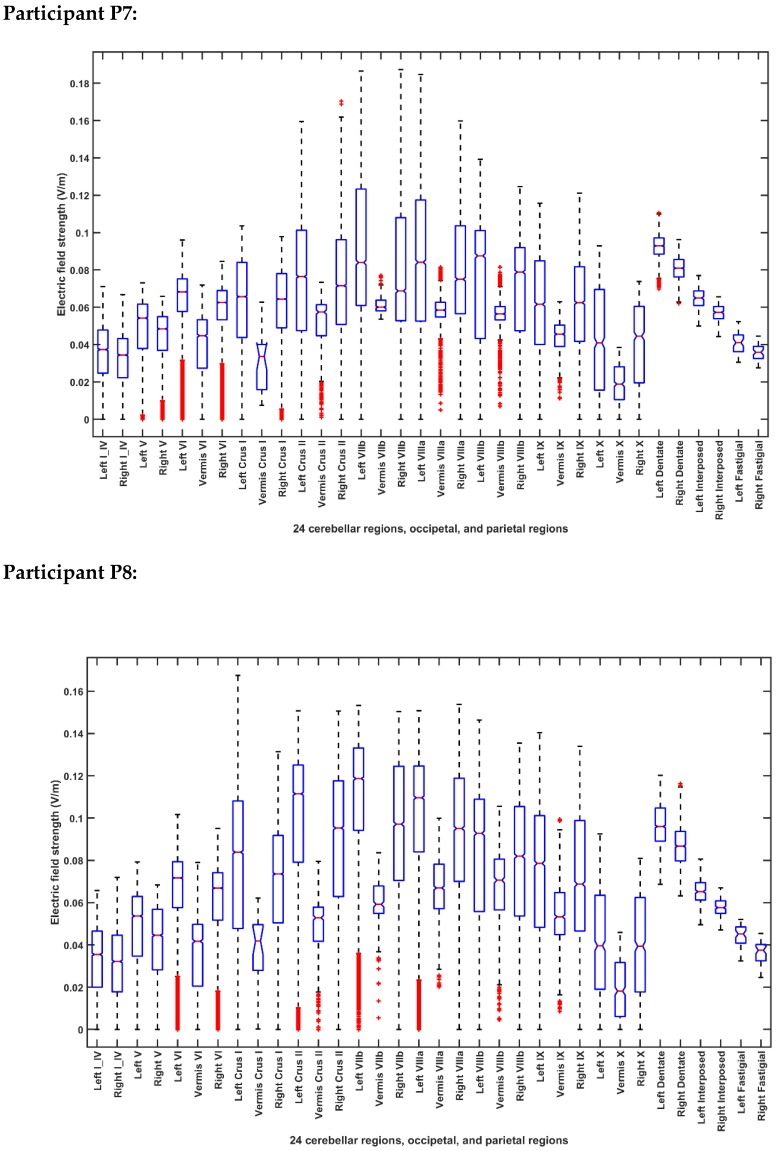

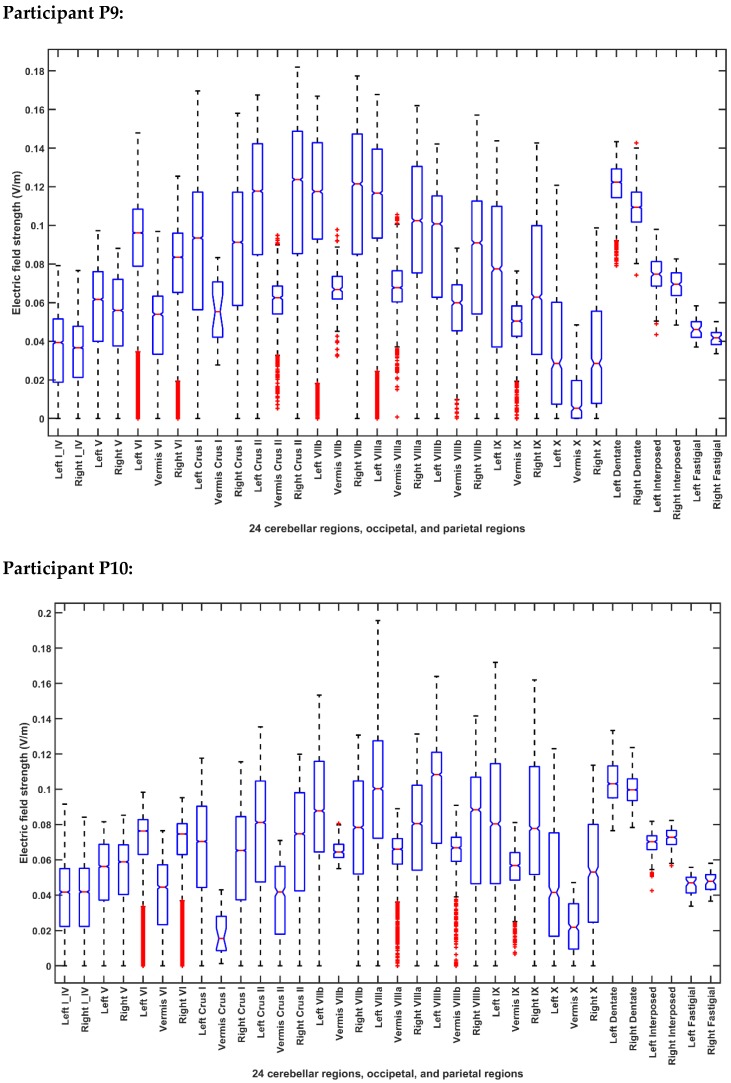
Boxplot of the lobular electric field distribution for the 10 post-stroke patients for the Exx7–Exx8 montage for case 2 (optimization for bilateral lobules VII–IX).

**Figure 7:**
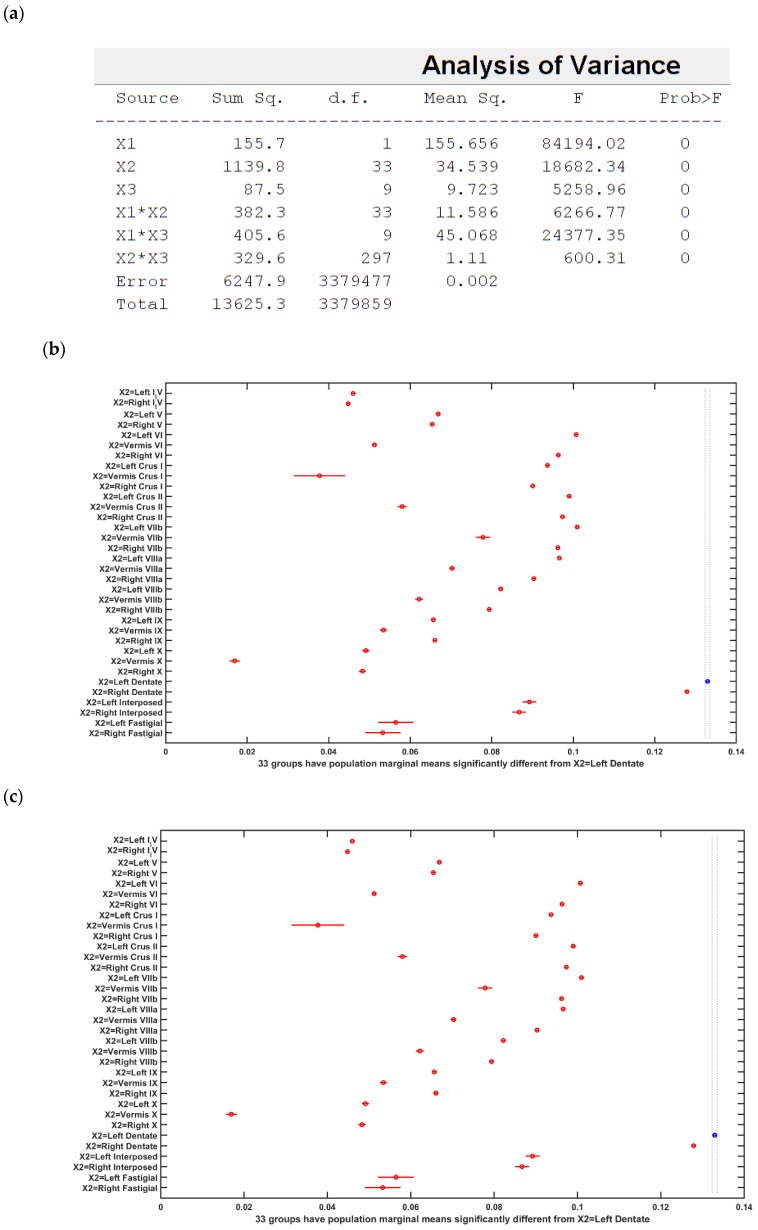

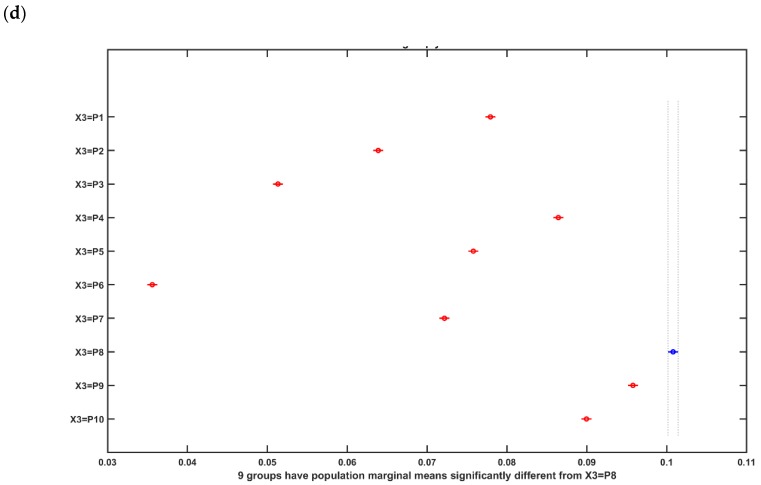
(**a**) ANOVA table: Three-way ANOVA for the factors of interest–montages (X1), brain regions (X2), subject (X3), and their interactions–all found significant. (**b)** Results of the multiple comparison test of the population marginal means between the PO9h–PO10h montage and the Exx7 – Exx8 montage (X1). (**c**) Results of the multiple comparison test of the population marginal means of different brain regions (X2). The dentate nuclei were exposed to the highest electric field strength (>0.12 V/m) across montages (X1) and subjects (X3). (**d**) Results of the multiple comparison test of the population marginal means of different subjects (X3). Subject P8 was exposed to the highest electric field strength (>0.1 V/m) across montages (X1) and brain regions (X2). Source: Source of variability;.Sum Sq.: Sum of Squares due to each source; d. f.: Degrees of freedom associated with each source; Mean Sq.: Mean Square for each source, which is the ratio Sum Sq. /d. f. F: statistics which is the ratio of the mean squares.

**Figure 8:**
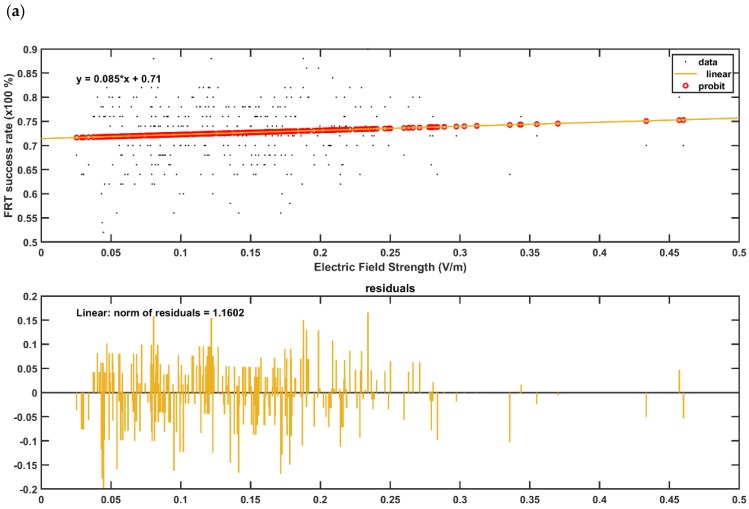

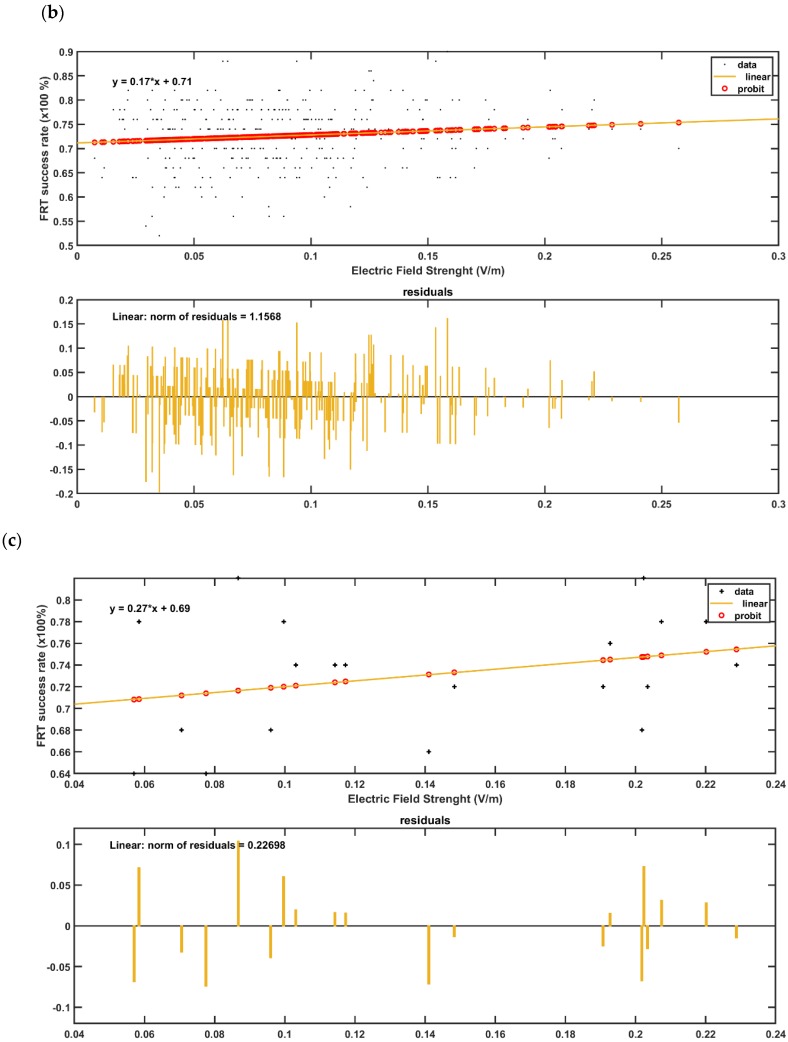

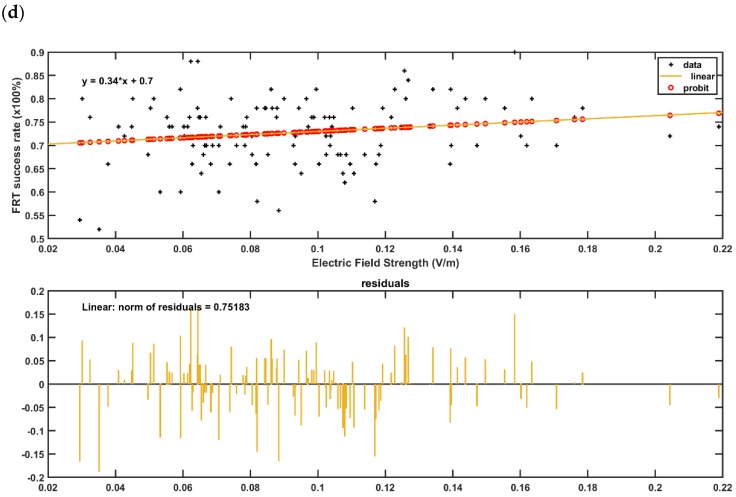
(**a**,**b**) GLM model (with probit link) for the post-intervention FRT success rate (%) with the lobular maximum electric field strength as the predictor in (**a**), and with the lobular median electric field strength as the predictor in the (**b**). The plot shows the observed and estimated the FRT success rate (%) versus the predictor values as well as the residuals where the FRT success rate (%) found to be more sensitive to lobular median electric field strength (slope 0.17—Figure 8b) than the lobular maximum electric field strength (slope 0.09—Figure 8**a**). (**c**,**d**) GLM model (with probit link) for the post-intervention FRT success rate (%) with the median electric field strength in dentate nuclei as the predictor in the **c**), and with the median electric field strength in the bilateral cerebellar leg area lobules as the predictor in the (**d**). The plot shows the observed and estimated the FRT success rate (%) versus the predictor values as well as the residuals where the FRT success rate (%) found to be more sensitive to the leg area lobular median electric field strength (slope 0.34—Figure 8d) than the dentate nuclei median electric field strength (slope 0.27—Figure 8c).

Figure 5 and Figure 6 present the boxplot of the electric field distribution for the 10 post-stroke patients for the PO9h–PO10h montage for case 1 (optimization for dentate nuclei) and for the Exx7–Exx8 montage for case 2 (optimization for bilateral lobules VII–IX) respectively. PO9h–PO10h montage for case 1 was optimized for the dentate nuclei which led to a higher electric field strength at the dentate nuclei as expected. However, PO9h–PO10h montage also led to an overall higher electric field strength at the bilateral leg lobules VII-IX when compared to the Exx7–Exx8 montage for the same stimulation current (2 mA). PO9h–PO10h montage also led to electric field spillover to the non-cerebellar occipital and parietal regions. Figure 7a shows three-way ANOVA results for the factors of interest–subjects, brain regions, montages, and their interactions which were all significant. Figure 7b shows the multiple comparisons of the population marginal means between the PO9h–PO10h montage and the Exx7–Exx8 montage which was found to be significantly (alpha = 0.05) different from each other. Figure 7c shows the multiple comparison test of the population marginal means of different brain regions (X2) where the dentate nuclei were exposed to a significantly (alpha = 0.05) higher electric field strength (>0.12 V/m) when compared to other brain regions across montages (X1) and subjects (X3). Figure 7d shows the multiple comparison test of the population marginal means of different subjects (X3) where the subjects P8-P10 were exposed to the significantly (alpha = 0.05) higher electric field strength when compared to other subjects across montages (X1) and brain regions (X2). Pre-intervention baseline equivalence of the FRT success rate (%) between the two repeated-measure counter-balanced crossover study groups was confirmed by the Wilcoxon rank-sum test that gave a p-value of 0.5216 so there was not enough evidence (5% significance level) to reject the null hypothesis of equal medians. The post-intervention FRT success rate (%) between the two repeated-measure counter-balanced crossover study groups gave a *p*-value of 4.6635 × e^−5^ so the null hypothesis of equal medians was rejected. Since the electric field, $\vec{E}$, distribution, as well as the post-intervention FRT success rate (%), significantly varied so a GLM model (with probit link) was fitted to the independent variable, FRT success rate (%), as the *p* × 1 behavioral outcome matrix, B, from the FRT study. Figure 8 shows that a probit fit is comparable to a linear fit along with its linear regression residuals. The top panel of Figure 8 shows the GLM model (with probit link) results where the lobular maximum electric field strength is the predictor in Figure 8a and the lobular median electric field strength is the predictor in Figure 8b. FRT success rate (%) was found to be more sensitive to the lobular median electric field strength than the lobular maximum electric field strength, slope 0.17 versus 0.09. Also, the bottom panel of Figure 8 shows the GLM model (with probit link) results where the median electric field strength in the dentate nuclei is the predictor in the Figure 8c and the lower-limb area lobular median electric field strength is the predictor in the Figure 8d. Here, the FRT success rate (%) was found to be more sensitive to the lower-limb representations lobular median electric field strength than the dentate nuclei median electric field strength, slope 0.34 versus 0.27.

## 4. Discussion

In this pilot study on 5 stroke survivors, ctDCS of the dentate nuclei facilitated greater target reaches during FRT when compared to bilateral cerebellar lower-limb representations ctDCS. Inter-subject variability in the electric field strength at the cerebellum, as shown in Figure 7d, is expected due to the “one-size-fits-all” approach taken in this preliminary study. Nevertheless, Figure 8 shows that the FRT success rate (%) was positively related to the electric field strength at the cerebellum. Here, the FRT success rate (%) was found to be more sensitive to the lower-limb representations lobular median electric field strength than the dentate nuclei median electric field strength. Therefore, a higher electric field strength at the lower-limb representations of the cerebellum is postulated to be responsible for the improvements found during our VR-based based target reaching task where ctDCS montage for the dentate nuclei (PO9h–PO10h) resulted in a significantly (alpha = 0.05) higher electric field strength when compared to the ctDCS montage for the bilateral lower-limb representation of the cerebellum (Exx7–Exx8) for the same stimulation current (2 mA) as shown in the Figure 7b. Electric field strength due to ctDCS montage for the dentate nuclei (PO9h–PO10h) reached not only the dentate nuclei but also reached lobules Crus I and Crus II (see Figure 4d ,e), and even resulted in a higher electric field strength at the cerebellar lower-limb representations when compared to the ctDCS montage for the bilateral lower-limb representations of the cerebellum (Exx7–Exx8) for the same stimulation current (2 mA)—see Figure 4, Figure 5 and Figure 6. This is due to a different objective function used to optimize ctDCS montage for the dentate nuclei versus that for the bilateral lower-limb representations of the cerebellum. The optimal ctDCS montage for the bilateral lower-limb representations of the cerebellum aimed for the uniform electric field in the bilateral cerebellar lobules VII-IX whereas the optimal ctDCS montage for the dentate nuclei aimed for maximum electric field strength. Here, the ctDCS montage for the bilateral lower-limb representations of the cerebellum (Exx7–Exx8) will require a higher stimulation current to reach comparable electric field strength at the lower-limb representations of the cerebellum. Also, this pilot study was limited by “one-size-fits-all” ctDCS montage for the dentate nuclei as well as the bilateral lower-limb representations of the cerebellum. In future clinical studies, subject-specific ctDCS montages need to be tested for post-stroke balance training.

The proposed deep ctDCS targeting the dentate nuclei were found to be painless by all the 5 subjects where a weak direct current (= 2 mA) was delivered through a 2 cm diameter saline-soaked sponge electrode overlying the cerebellum. Skin irritation was found in one subject which can be due to a relatively high current density of 0.635 mA/cm^2^. Larger electrode size can ameliorate this issue in subjects with sensitive skin. Overall, bipolar bilateral ctDCS of the dentate nuclei performed better than the bipolar bilateral ctDCS of the cerebellar lower-limb representations for the same 2mA stimulation current where extensive reciprocal connectivity between the dentate nucleus and the wide swaths of cerebral cortex can affect motor as well as cognitive function [12]. Figure 4 shows that conventional ctDCS montages, e.g., Celnik, Manto, and Extracephalic, all affected the dentate nuclei so the functional effects due to these conventional ctDCS montages should be investigated not only based on its effects by polarizing Purkinje cells [13] but also based on its effects on the dentate nuclei. Also, the electric field effects of the Manto ctDCS montage were primarily focused on the cerebellar lower-limb representations which were found to be comparable to our ctDCS montage (Exx7–Exx8 montage)—see Figure 4b,e. We also found that electric field effects of our ctDCS montage for the dentate nuclei (PO9h–PO10h) reached lobules Crus I and Crus II (see Figure 4d) which can have beneficial cognitive effects by polarizing Purkinje cells. Nevertheless, the effectiveness of ctDCS of dentate nuclei needs to be tested as an adjuvant treatment to VR-based balance/weight-shifting training since the final goal is to improve functional outcomes. This pilot study was limited by a heterogeneous small sample size due to convenience sampling which consisted of 10 males within a large age range from 28 years to 59 years. Therefore, a larger clinical study is necessary to statistically confirm the effectiveness of ctDCS of the dentate nuclei with objective balance assessments.

During balance rehabilitation, an objective assessment of the balance and posture during functional reach tasks (FRT) or cued weight-shifting will require optical motion analysis technology that can provide a sensitive measure. However, marker-based systems (e.g., VICON, UK) are too expensive for monitoring in a community setting not only in developing countries but also in developed countries including the USA. Therefore, we developed marker-less time-of-flight systems [16,26,63,64], including the low-cost (<$150) Microsoft Kinect sensor (developed for video gaming), that are increasingly used for motion capture due to its lower costs [65]. Also, postural sway based on the center of pressure (CoP) is important for balance assessment; and the Wii Balance Board (WiiBB) has demonstrated good test-retest reliability (ICC = 0.66–0.94) and construct validity when benchmarked against laboratory-grade force platforms (ICC = 0.77–0.89) [66]. In principal accordance, we used low-cost (<$150) WiiBB for standing balance tests where cursor tracking in the virtual reality (VR) using CoP has been implemented and tested for usability [23]. This HMI has also been tested under an operant conditioning paradigm for balance training [67] where the cursor (a VR object) controller is designed such that it is less challenging to control using paretic leg to reach the cued VR targets, and this innovative approach can ameliorate learned non-use of the paretic leg by encouraging its increased use during adaptive VR-based weight-shifting tasks [67]. We postulate that operant conditioning can lead to learned internal representations and response to stimuli that can be facilitated with adjuvant treatment with ctDCS [68,69]. Here, VR-environment for FRT is postulated to be motivating thereby improving the therapy effects (Gil-Gómez et al., 2011). Indeed, systematic review and meta-analysis based on forty-three randomized controlled trials have shown that balance capacities can be improved by well-targeted exercise therapy programs, specifically, balance and/or weight-shifting training, in the chronic phase after stroke [70]. Furthermore, another systematic review and meta-analysis based on literature searches in databases including PubMed, Embase, MEDLINE, and Cochrane Library by de Rooij et al. [71] showed that VR training is more effective than balance training without VR for improving balance ability in patients with stroke. Also, a systematic review on feasibility and effectiveness based on literature searches in five databases including PubMed and the Cochrane Library [72] showed that VR can increase motivation allowing longer and more training sessions in community-dwelling stroke survivors. Nevertheless, there exists very little evidence about interventions other than exercises, e.g., ctDCS, that can improve post-stroke standing balance function [73].

To investigate post-stroke standing balance function, CoP trajectories during cued weight shifts in different directions during FRT [23] can elucidate ideomotor apraxia (IMA) found in our subjects, which may contribute to patients overall day-to-day motor disability [74]. IMA of lower limbs has rarely been investigated systematically [75] even though it has high relevance for maintaining independence in daily life activities [76]. Furthermore, IMA is one the earliest disturbances in Alzheimer′s disease [77] where disruption of the cerebrocerebellar network has been hypothesized based on the executive cluster of the cerebellar cognitive affective syndrome (CCAS) [59]. We recently presented ctDCS for healthy aging [78] where bipolar bilateral ctDCS can be a low-cost intervention that needs to be validated using a prospective, randomized, placebo-controlled, double-blinded, clinical study. Also, according to the majority of studies in the literature, limb apraxia in right-handed stroke patients is a disorder that typically occurs in 30–50% of the patients with left hemisphere damage and 0 to 30% in patients with right hemisphere damage [76]. Here, post-stroke IMA patients with left posterior parietal and/or premotor cortex lesions but intact cerebellum can be suitable for ctDCS. Therefore, we are investigating the feasibility of our mobile VR-based balance training in conjunction with bilateral ctDCS in the right lower limb post-stroke IMA with left hemisphere damage [75]. IMA is also an area of scientific significance for our operant conditioning approach to balance training [67] where one can learn how the normal human praxis system improves in IMA during adaptive balance training [67]. This is postulated due to IMA’s cerebellar component related to cardinal motor deficits which are thought to arise from damage to the cerebrocerebellar network communicating internal representations for actions. Here, an early operant conditioning approach to balance training [67] in a community setting after stroke may be crucial for recovery through learning and experience [79]. Recovery through balance training [67] is crucial since falls are more likely in the apraxias [80], and the severity of apraxia predicts the rehabilitation success for patients with hemiplegia [76]. In principal accordance, an augmented mobile VR interface can allow remote delivery of new VR balance training games to keep the motivation for home-based intervention. To address usability issues with individualized ctDCS montage, we have developed an innovative low-cost washable neoprene cap with subject-specific stitched saline-soaked electrodes that can be worn during balance training [20]. Such a home-based upper-limb training approach has also been proposed by de Winckel and colleagues [22], however, our approach is novel for lower limb balance training.

Our optimization approach for a minimal set of electrodes for home-based tDCS is based on an open-source computational pipeline [7] that aimed to keep the bilateral ctDCS electric field limited to the cerebellum and away from the cerebral areas that were lesioned in our stroke subjects. Here, first, a reduced set of electrodes (see Figure 3) relevant for focal stimulation of the cerebellum were identified in the stroke subjects for the optimization of a “one-size-fits-all” bipolar electrode montage for ctDCS, as discussed in the Methods section. This two-step process to identify a “one-size-fits-all” bipolar electrode montage to target cerebellar lobules and nuclei is postulated to be more practical in low-resource home-based or community-based settings constrained by a lack of computing power and high-quality neuroimaging data. However, our innovation in optimizing the lobular electric field for patient-relevant functional outcome and neuroplastic effects in stroke survivors is also important for patient-specific dosing based on MRI data that may reduce inter-individual variability [81]. Here, optimization based on the relevant component of the electric field [81] needs to be verified for different cerebellar lobular targets, including the molecular layer, the granule cell layer and the Purkinje cell layer, since different ctDCS electrode montages can affect different parts of the cerebellum (and cerebellar circuit) [7] leading to different functional outcomes and neuroplastic effects. For example, anodal ctDCS using Celnik montage [13] affected the adaptation rate of spatial but not temporal elements of walking where the spatial adaptation was postulated to be related to pontocerebellum stimulation [10]. Our open-source computational modeling pipeline [7] confirmed that the magnitude of the electric field for Celnik montage [13] primarily targeted the pontocerebellum as postulated in the experimental paper by Jayaram et al. [10]. The innovation lies not only in the ability to optimize the lobular electric field in the cerebellum [7] but also in the augmented approach to address functional heterogeneity [58] based on the outcomes from a task battery. Here, post-stroke deficits can cover multiple task domains that can be elucidated with multi-domain behavioral experiments (e.g., FRT balance function, hand function, gait function, cognitive function) to develop an appropriate objective function for ctDCS optimization that addresses cerebellar multiple functionalities [58]. Here, optimization of the lobular electric field in the cerebellum [7] is challenging without subject-specific neuroimaging guided head modeling due to the extreme folding of the cerebellar cortex. We also postulate for future studies that ctDCS optimization needs to be based on the mapping to universal cerebellar computations (e.g., comparator function) [58] that can ameliorate deficits in multiple task domains [58].

Neurorehabilitation service delivery at homes and in the community settings can incorporate mobile-health based approaches to low-cost neurotechnologies that are tailored to an individual health condition as identified based on WHO International Classification of Functioning (ICF) [82]. Here, functional optimization of ctDCS will require a large patient outcome dataset possible using big data mobile-health (mHealth) approaches that also requires sustainable and multi-professional rehabilitation systems, including the provision of services to the rural population. This was investigated by a randomized controlled trial on family-led rehabilitation after stroke in India (ATTEND) [83]. In the ATTEND trial, regular stroke rehabilitation services provided by family caregivers were found not effective even after structured training including information provision, joint goal setting, carer training, and task-specific training [83]. ATTEND trial suggested investigation of the effects of task shifting to health-care assistants or team-based community care that necessitated telerehabilitation strategies due to the scarcity of trained professionals in India. Furthermore, telestroke model in India for thrombolysis in acute ischemic stroke showed that smartphone-based telestroke services may be a much cheaper alternative to video-conferencing-based telestroke services and are more portable with less technical glitches [84]. India is ripe for the assessment of the feasibility and usability of telemedicine approaches not only in acute stroke [84] but also in chronic stroke. Tele-rehabilitation is also justified since functional improvements have been found to be equal for telerehabilitation and virtual reality (VR)-based training when compared to a similar intervention with therapist-supervision in the clinic [72]. Therefore, we propose testing of the effectiveness of a low-cost neurotechnology platform [23] for remote (smartphone-based) patient care and monitoring through the hub and spoke model (HSM) of telemedicine that is not only necessary to create a large patient outcome dataset but is also crucial to meet the growing needs of stroke survivors in India [85,86]. In the HSM of neurorehabilitation, the service delivery assets into a network consist of an anchor establishment (hub) which will offer a full array of services, complemented by secondary community-based establishments (spokes) that can offer local neurorehabilitation service arrays, routing patients needing more intensive services to the hub for treatment. Here, the feasibility of an online assessment document called ‘Rehabilitation Problem-Solving Form’ (RPS-Form) [87] was shown by us in a preliminary study in India to monitor patient’s response to a short-duration moderate-intensity neurostimulation therapy by assessing all the ICF components. Here, our innovative online RPS-Form captures patients’ perspective that has been shown in our preliminary study [87] to facilitate communications between the patient (at the spoke in HSM) and his/her multi-disciplinary rehabilitation team (at the hub in HSM) consisting of physiotherapists, occupational therapists, medical doctors, and rehabilitation engineers. Such multi-session neurotechnology intervention may be necessary for a community setting where post-stroke remote tDCS plus target tracking training has been shown feasible and usable for upper limb [22], however, the feasibility of remote delivery of ctDCS in conjunction with mobile VR-based balance training in a low resource community setting is unknown. Here, the feasibility and usability testing of remote delivery of ctDCS are crucial since usability issues could lead to user error that has the potential to compromise patient safety and negatively impact the quality of therapy and outcomes.

## 5. Conclusions

We developed and evaluated a rational approach to optimize deep ctDCS of the dentate nuclei and lower limb representations in the cerebellum for post-stroke balance rehabilitation. Our pilot study presented promising results on the beneficial effects of deep ctDCS on functional reach during a standing balance task in chronic stroke survivors. However, our clinical study in a low-resoure setting was limited by “one-size-fits-all” bipolar ctDCS montage as well as a small sample size.

## Figures and Tables

**Table 1:** Ten chronic (>6 months) stroke participants.

Participant ID	Age	Gender	Hemiplegic Side
P1 *	48 years	Male	LEFT
P2 *	38 years	Male	RIGHT
P3	35 years	Male	LEFT
P4 *	44 years	Male	RIGHT
P5	56 years	Male	RIGHT
P6	59 years	Male	RIGHT
P7	28 years	Male	LEFT
P8 *	50 years	Male	LEFT
P9	50 years	Male	LEFT
P10 *	32 years	Male	RIGHT

* starred participants completed the FRT study.

**Figure A2:**
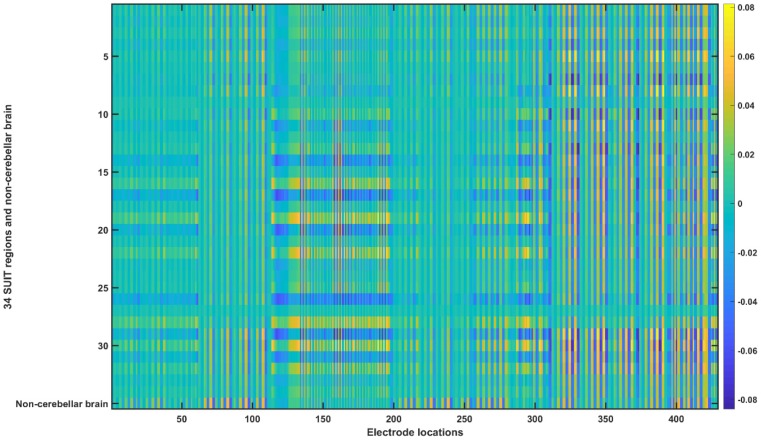
Ex transfer matrix for the head model from the 55–59 years old MRI template. The X-axis shows the electrode locations (refer Appendix B) and Y-axis shows the 34 SUIT regions (refer Appendix A) and non-cerebellar brain.

**Figure A5:**
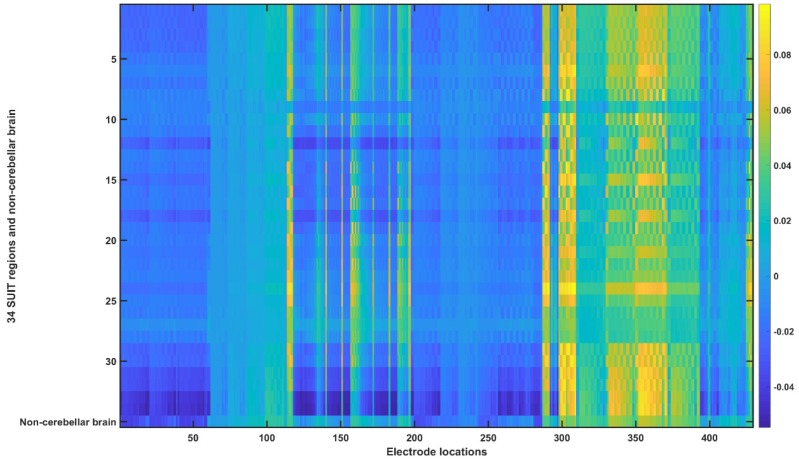
Ey transfer matrix for the head model from the 55–59 years old MRI template. The X-axis shows the electrode locations (refer Appendix B) and Y-axis shows the 34 SUIT regions (refer Appendix A) and non-cerebellar brain.

**Figure A8:**
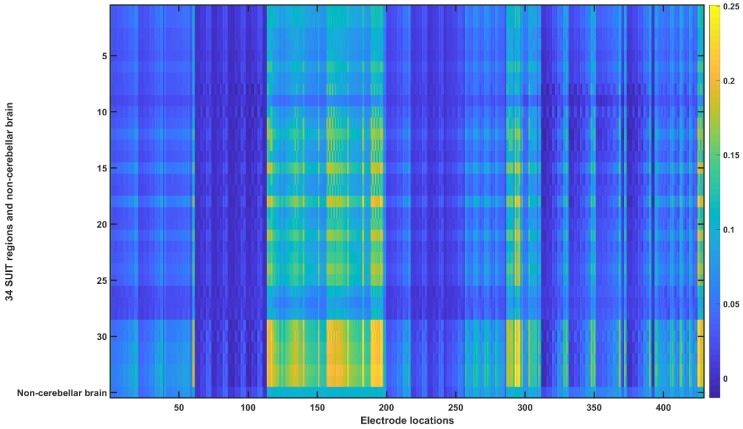
Ez transfer matrix for the head model from the 55–59 years old MRI template. The X-axis shows the electrode locations (refer Appendix B) and Y-axis shows the 34 SUIT regions (refer Appendix A) and non-cerebellar brain.

**Figure A11:**
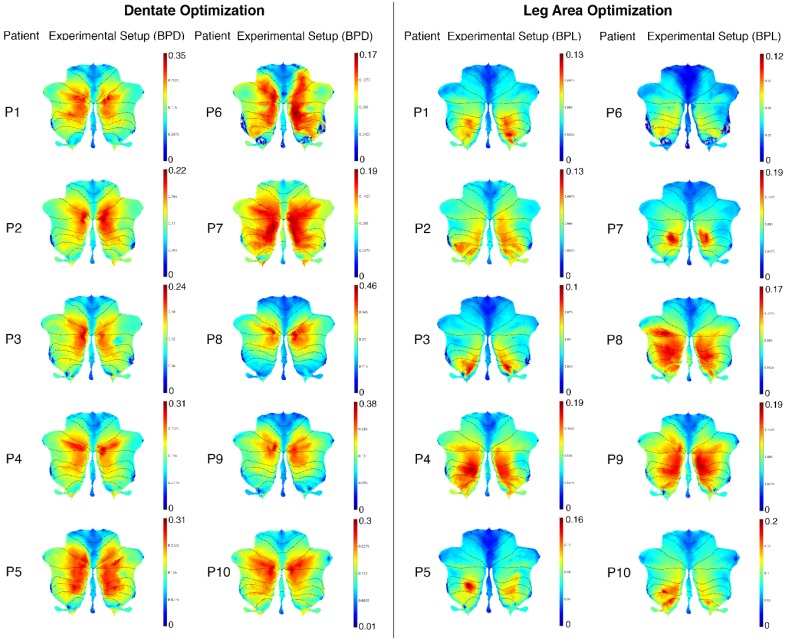
Post-stroke subject-specific SUIT flatmaps for all the 10 participants.

## Appendix A

**Table A1:** The 34 SUIT labels are.

1	Left I_IV	10	Right Crus I	19	Right VIIIa	28	Right X
2	Right I_IV	11	Left Crus II	20	Left VIIIb	29	Left Dentate
3	Left V	12	Vermis Crus II	21	Vermis VIIIb	30	Right Dentate
4	Right V	13	Right Crus II	22	Right VIIIb	31	Left Interposed
5	Left VI	14	Left VIIb	23	Left IX	32	Right Interposed
6	Vermis VI	15	Vermis VIIb	24	Vermis IX	33	Left Fastigial
7	Right VI	16	Right VIIb	25	Right IX	34	Right Fastigial
8	Left Crus I	17	Left VIIIa	26	Left X		
9	Vermis Crus I	18	Vermis VIIIa	27	Vermis X		

## Appendix B

**Table A2:** 429 electrode locations (including fiducials and reference electrodes) with the X, Y, Z for 55–59 years head model are.

No.	Location	x	y	z	No.	Location	x	y	z
1	Fp1	−22.5592	82.90488	13.87207	216	P5h	−62.6435	−55.417	48.50705
2	Fpz	3.842296	87.03616	17.49313	217	P3h	−44.5991	−62.2382	71.54874
3	Fp2	28.52678	83.55578	13.67529	218	P1h	−15.6894	−68.0947	83.5882
4	AF7	−41.9976	71.24048	12.12234	219	P2h	19.79522	−68.738	83.61536
5	AF5	−34.6647	75.10938	25.48365	220	P4h	49.80881	−62.9955	71.23532
6	AF3	-25.3904	78.60305	35.73096	221	P6h	67.89475	-54.8502	47.65088
7	AF1	-11.941	79.80824	43.49709	222	P8h	73.34005	−48.4089	22.94698
8	AFz	2.700881	80.83209	45.4243	223	P10h	74.35319	−44.3501	−5.70373
9	AF2	18.38834	80.3016	43.27522	224	PO9h	−55.5975	−70.1245	−6.82361
10	AF4	32.66851	78.83771	35.49923	225	PO7h	−54.5191	−76.0335	19.40764
11	AF6	41.63063	75.41405	25.66882	226	PO5h	−45.1531	−82.1967	35.4382
12	AF8	47.49697	72.00578	13.61991	227	PO3h	−29.3346	−88.0844	47.25826
13	F9	−55.94	50.26538	−18.4116	228	PO1h	−8.08273	−90.9205	53.45393
14	F7	−56.1724	53.03701	11.21066	229	PO2h	13.28204	−91.068	52.90984
15	F5	−50.8744	56.21025	31.86567	230	PO4h	32.52896	−88.4084	47.14226
16	F3	−39.0952	59.77218	50.02259	231	PO6h	50.25244	−81.431	35.07223
17	F1	−20.1033	63.803	63.89388	232	PO8h	59.58925	−74.9128	19.71736
18	Fz	2.99712	65.08257	69.42619	233	PO10h	61.64832	−69.6594	−5.77207
19	F2	27.36842	63.47771	64.33418	234	O1h	−16.0684	−100.448	16.96381
20	F4	45.65995	60.44671	50.90544	235	O2h	20.76301	−100.285	15.9655
21	F6	57.03421	57.12775	33.22234	236	I1h	−11.759	−96.2474	−22.6608
22	F8	62.18449	53.92647	12.13087	237	I2h	16.51372	−96.2668	−21.869
23	F10	61.52017	51.90641	−17.3684	238	AFp7	−32.0664	78.3587	13.15924
24	FT9	−66.2371	32.61463	−20.9561	239	AFp5	−25.9275	80.75012	21.30455
25	FT7	−65.3194	31.50805	11.14653	240	AFp3	−19.2884	82.73908	28.30598
26	FC5	−62.0899	32.15929	37.65389	241	AFp1	−8.28792	84.68655	31.39793
27	FC3	−49.3727	33.4715	61.182	242	AFpz	3.526845	85.00818	32.90711
28	FC1	−26.791	37.68753	80.24023	243	AFp2	14.7652	84.92751	31.13003
29	FCz	4.253833	38.91969	87.41366	244	AFp4	24.73355	83.55391	28.15581
30	FC2	33.31507	37.83103	80.37218	245	AFp6	33.52511	80.75925	22.74153
31	FC4	56.8832	33.78278	61.13577	246	AFp8	37.81402	79.40772	12.13282
32	FC6	69.41998	31.88483	37.35075	247	AFF9	−48.1636	62.60542	−15.9273
33	FT8	72.12511	31.09664	11.29649	248	AFF7	−49.5452	63.06089	11.20612
34	FT10	72.28736	31.96451	−21.5403	249	AFF5	−43.4247	66.72266	28.63601
35	T9	−68.4998	8.249681	−22.4725	250	AFF3	−32.7211	69.92124	43.53524
36	T7	−71.3853	5.05698	11.08433	251	AFF1	−16.6866	72.82261	53.58292
37	C5	−69.229	3.884095	42.42783	252	AFFz	4.288832	74.71173	57.53587
38	C3	−57.747	3.717831	67.76955	253	AFF2	22.84423	73.16779	54.42189
39	C1	−31.5817	4.967477	89.48766	254	AFF4	38.48791	71.41055	43.94503
40	Cz	3.456495	5.329642	99.52775	255	AFF6	50.03043	67.02973	29.6401
41	C2	38.09238	5.82291	88.99616	256	AFF8	55.65077	63.6887	11.91936
42	C4	64.26701	4.369621	67.62971	257	AFF10	54.26406	62.65546	−16.8431
43	C6	75.43402	6.505909	42.33944	258	FFT9	−62.323	41.66684	−20.9349
44	T8	77.38602	6.530228	9.717833	259	FFT7	−61.3234	42.58761	10.88625
45	T10	74.50288	7.909821	−22.3627	260	FFC5	−57.0247	44.441	34.49869
46	TP7	−73.5016	−21.5145	10.67297	261	FFC3	−44.6976	46.61251	56.76297
47	CP5	−71.524	−24.3614	42.38637	262	FFC1	−24.2908	51.55225	72.34157
48	CP3	−61.6892	−27.3726	69.56094	263	FFCz	3.465133	54.22735	79.14475
49	CP1	−33.5793	−28.5314	92.61813	264	FFC2	31.00001	51.91758	72.89468
50	CPz	3.611006	−29.6046	102.548	265	FFC4	51.2962	48.01261	57.39484
51	CP2	40.46755	−28.5723	92.68565	266	FFC6	64.18185	44.11128	35.40215
52	CP4	67.15958	−26.2549	70.12574	267	FFT8	68.26	42.44242	10.99613
53	CP6	77.5511	−23.1143	40.75406	268	FFT10	67.77986	42.6417	−21.5682
54	TP8	79.52268	−20.9995	9.849888	269	FTT9	−68.2673	19.54876	−20.5511
55	P9	−67.2474	−45.3603	−21.1713	270	FTT7	−68.4895	19.30654	10.96369
56	P7	−68.778	−46.0653	11.4691	271	FCC5	−66.1508	19.10656	40.53259
57	P5	−66.2887	−52.513	36.08666	272	FCC3	−54.2756	17.88662	64.73584
58	P3	−54.9608	−59.6442	60.7231	273	FCC1	−29.3821	20.95851	86.31815
59	P1	−29.4783	−66.3772	79.33228	274	FCCz	3.958818	22.00332	94.121
60	Pz	1.16076	−67.0461	86.85532	275	FCC2	35.52612	22.59816	85.42201
61	P2	35.68975	−66.3795	79.29573	276	FCC4	60.75797	19.88735	64.52368
62	P4	60.31538	−59.8885	59.73956	277	FCC6	72.32896	21.06874	40.94543
63	P6	71.43842	−51.4954	35.46394	278	FTT8	75.15998	19.07234	11.70313
64	P8	74.42983	−46.1598	10.89193	279	FTT10	74.18301	19.9038	−19.5862
65	P10	73.05866	−44.5554	−22.0247	280	TTP7	−73.2764	−8.59877	11.85397
66	PO9	−55.4014	−66.2734	−22.0704	281	CCP5	−71.2532	−9.33826	42.32324
67	PO7	−57.4879	−72.0804	11.77603	282	CCP3	−61.0012	−11.1267	68.91452
68	PO5	−50.2619	−79.2832	27.65109	283	CCP1	−33.6162	−11.7103	91.75061
69	PO3	−38.3293	−85.4101	41.33633	284	CCPz	3.522455	−12.3699	102.5344
70	PO1	−18.2358	−90.253	50.93382	285	CCP2	40.14838	−11.1746	92.32234
71	POz	3.252028	−91.057	54.66248	286	CCP4	66.77457	−10.8469	70.0064
72	PO2	24.01926	−90.0057	50.48114	287	CCP6	77.44537	−8.49465	41.47523
73	PO4	42.66196	−85.4391	40.63985	288	TTP8	79.36731	−7.57537	11.90171
74	PO6	55.28989	−78.3901	27.5132	289	TPP9	−69.0679	−26.4722	−22.8065
75	PO8	62.37442	−71.6847	11.74387	290	TPP7	−72.1635	−32.9648	10.49897
76	PO10	60.72315	−67.1283	−20.8686	291	CPP5	−70.2081	−37.2139	39.19454
77	O1	−32.7194	−94.0121	14.27106	292	CPP3	−60.0331	−43.4354	65.72448
78	Oz	3.040962	−102.261	17.13088	293	CPP1	−33.7106	−48.8915	88.78305
79	O2	38.5953	−93.1054	13.28896	294	CPPz	3.471033	−50.1578	96.55543
80	O9	−31.4911	−90.3528	−22.2667	295	CPP2	41.27761	−47.5401	88.19879
81	O10	35.9837	−91.066	−21.1492	296	CPP4	65.71957	−43.0657	65.24334
82	I1	−31.4911	−90.3528	−22.2667	297	CPP6	75.38837	−37.5137	39.31531
83	I2	35.9837	−91.066	−21.1492	298	TPP8	78.0984	−32.6421	11.23656
84	AFp9h	−33.4931	79.64136	−1.93019	299	TPP10	77.33778	−26.388	−23.5285
85	AFp7h	−29.075	79.48131	17.76607	300	PPO9	−62.4592	−53.9455	−21.5535
86	AFp5h	−22.3499	82.01069	25.16756	301	PPO7	−64.3126	−60.0043	11.86185
87	AFp3h	−14.4406	83.74335	30.16004	302	PPO5	−60.4425	−66.1359	31.64876
88	AFp1h	−2.90148	85.04022	31.85801	303	PPO3	−47.0598	−74.4885	51.85476
89	AFp2h	9.675535	85.09084	32.41395	304	PPO1	−25.6341	−79.8341	65.64269
90	AFp4h	19.4288	84.61374	29.56817	305	PPOz	2.544747	−82.1084	69.75008
91	AFp6h	28.54921	82.69922	24.88188	306	PPO2	30.92945	−80.0041	65.53837
92	AFp8h	34.67982	80.53532	17.90698	307	PPO4	52.82137	−73.5944	51.12067
93	AFp10h	39.61572	79.72829	−3.10175	308	PPO6	65.23055	−65.1262	31.95901
94	AFF9h	−50.3731	65.10702	−5.09855	309	PPO8	69.65572	−58.325	10.88854
95	AFF7h	−47.2197	64.64239	21.10871	310	PPO10	67.54266	−54.4292	−22.6903
96	AFF5h	−38.0659	69.20643	35.60582	311	POO9	−46.5394	−78.0818	−22.3323
97	AFF3h	−25.6713	71.1931	49.07541	312	POO7	−47.1039	−83.4091	13.77876
98	AFF1h	−6.32591	73.72855	57.30378	313	POO5	−38.0156	−90.045	22.25939
99	AFF2h	13.28156	74.02155	57.3758	314	POO3	−26.5354	−95.0087	30.24876
100	AFF4h	32.14301	72.00444	49.46066	315	POO1	−11.3729	−97.5071	35.97657
101	AFF6h	45.62772	68.72171	37.0618	316	POOz	1.421684	−98.1703	36.50932
102	AFF8h	52.96668	66.07579	20.63462	317	POO2	18.62236	−97.2628	34.81373
103	AFF10h	56.20926	65.41907	−5.3522	318	POO4	33.08083	−93.8825	29.92061
104	FFT9h	−62.2082	42.18876	−7.02438	319	POO6	43.59314	−89.0643	22.69428
105	FFT7h	−60.208	43.71072	22.80203	320	POO8	52.61583	−82.9258	13.22738
106	FFC5h	−52.1008	45.21527	46.08334	321	POO10	52.20508	−78.4769	−21.0116
107	FFC3h	−35.2463	49.01726	65.63649	322	OI1	−33.6804	−92.1769	−4.83753
108	FFC1h	−10.0519	51.67205	78.71602	323	OIz	2.777775	−101.21	−5.77148
109	FFC2h	16.63637	52.14783	78.93129	324	OI2	38.28328	−92.2741	−4.78841
110	FFC4h	41.65304	50.35553	66.03575	325	T3	−71.3853	5.05698	11.08433
111	FFC6h	58.85195	46.52834	46.21732	326	T5	−68.778	−46.0653	11.4691
112	FFT8h	67.11803	42.89774	24.38205	327	T4	77.38602	6.530228	9.717833
113	FFT10h	68.23584	42.95902	−6.70591	328	T6	74.42983	−46.1598	10.89193
114	FTT9h	−68.3143	20.6105	−6.39526	329	Exz	3.413347	−94.1549	−34.1897
115	FTT7h	−68.134	19.9404	27.09219	330	Ex1	−8.96197	−94.2189	−34.75
116	FCC5h	−62.2303	17.18796	52.15804	331	Ex2	13.74652	−94.1877	−33.868
117	FCC3h	−42.1513	19.75226	77.24973	332	Ex3	−36.2817	−82.5359	−38.5457
118	FCC1h	−12.9036	22.0511	91.96693	333	Ex4	43.2247	−80.4344	−40.514
119	FCC2h	19.41887	22.7243	91.5395	334	Ex5	−45.9755	−71.6576	−39.8934
120	FCC4h	49.69143	20.55158	76.14634	335	Ex6	51.25343	−72.2088	−40.2305
121	FCC6h	68.2901	18.7172	53.31686	336	Ex7	−53.701	−60.5663	−40.0393
122	FTT8h	75.10453	19.11036	25.89622	337	Ex8	60.58237	−59.2705	−39.1244
123	FTT10h	74.3296	21.48304	−7.01454	338	Ex9	−59.7161	−48.2031	−38.4779
124	TTP7h	−73.2487	−9.19111	26.35322	339	Ex10	65.82136	−47.2179	−39.1501
125	CCP5h	−68.0965	−11.9187	56.38265	340	Ex11	−61.9403	−42.6414	−39.7219
126	CCP3h	−49.0741	−11.6074	82.11982	341	Ex12	68.00566	−42.5266	−39.2374
127	CCP1h	−15.7263	−11.6781	99.48219	342	Ex13	−65.1545	−22.0944	−31.4051
128	CCP2h	21.99551	−11.2966	99.28143	343	Ex14	69.53552	−21.2467	−32.9172
129	CCP4h	55.17046	−11.6881	82.76363	344	Ex19	−67.1288	8.122559	−35.3273
130	CCP6h	74.18729	−8.89512	55.26226	345	Ex20	72.7148	8.95946	−35.0052
131	TTP8h	79.55205	−7.03237	25.88526	346	Ex21	−65.7359	19.52943	−34.2457
132	TPP9h	−73.1609	−31.863	−6.42023	347	Ex22	71.67707	20.64119	−34.9331
133	TPP7h	−71.2541	−35.6643	25.71736	348	Ex23	−64.4187	30.91435	−34.9063
134	CPP5h	−66.9441	−40.7847	52.23498	349	Ex24	70.69261	30.84808	−34.6901
135	CPP3h	−48.5238	−46.0193	78.62906	350	Ex25	−61.0794	41.18943	−35.0183
136	CPP1h	−16.212	−48.3473	95.56354	351	Ex26	66.73764	41.93864	−34.4454
137	CPP2h	22.19478	−48.9157	95.74229	352	Ex27	−54.5623	51.50744	−34.1932
138	CPP4h	54.62639	−44.8678	78.57848	353	Ex28	60.44307	51.08272	−33.6067
139	CPP6h	72.4343	−40.4486	51.81738	354	Ex29	−46.6597	58.25651	−33.7596
140	TPP8h	77.38898	−35.5248	24.96803	355	Ex30	52.00837	59.01769	−33.3612
141	TPP10h	78.72811	−31.5109	−7.08444	356	Ex31	−37.3566	64.40849	−32.5878
142	PPO9h	−63.5017	−57.5402	−6.52086	357	Ex32	42.79809	64.87608	−33.3054
143	PPO7h	−63.334	−62.1435	22.93271	358	Ex33	−27.8098	69.11741	−31.0611
144	PPO5h	−54.6809	−70.9147	42.44857	359	Ex34	34.60232	69.6494	−30.5847
145	PPO3h	−37.0488	−78.1767	59.33378	360	Exxz	2.600347	−88.223	−53.4067
146	PPO1h	−11.9075	−81.2245	69.38043	361	Exx1	−14.7184	−87.6082	−53.7016
147	PPO2h	17.13343	−81.2939	69.21624	362	Exx2	12.11622	−88.1398	−53.8715
148	PPO4h	43.56473	−76.6983	59.87423	363	Exx3	−39.6464	−73.0199	−51.5006
149	PPO6h	60.5297	−69.1201	42.01922	364	Exx4	44.51721	−73.1659	−52.6129
150	PPO8h	67.73132	−61.8571	21.41818	365	Exx5	−49.3719	−59.3122	−53.2278
151	PPO10h	69.40595	−56.9527	−5.5405	366	Exx6	54.64212	−60.8592	−52.4478
152	POO9h	−46.5702	−81.4362	−6.27118	367	Exx7	−54.6454	−44.98	−54.0367
153	POO7h	−42.5362	−87.1419	17.76792	368	Exx8	59.84738	−49.264	−52.8618
154	POO5h	−32.906	−92.4566	26.75433	369	Exx9	−56.4254	−37.1207	−53.3956
155	POO3h	−18.1407	−97.0718	32.9983	370	Exx10	63.56102	−35.4075	−53.3598
156	POO1h	−4.35032	−98.1974	36.47809	371	Exx11	−57.4456	−29.9627	−53.944
157	POO2h	10.44098	−98.1735	35.64018	372	Exx12	64.68636	−31.6225	−53.0232
158	POO4h	24.9554	−96.0371	33.98659	373	Exx13	−59.97	−21.5577	−55.1901
159	POO6h	39.33543	−91.2763	26.13067	374	Exx14	66.54841	−22.0255	−55.3961
160	POO8h	48.66409	−85.8521	17.3463	375	Exx15	−62.8827	−8.8041	−51.3662
161	POO10h	51.90488	−81.7238	−3.61663	376	Exx16	69.73143	−8.80946	−51.6004
162	OI1h	−16.2967	−99.2963	−5.70751	377	Exx19	−62.6843	10.32494	−49.7987
163	OI2h	19.77029	−99.3035	−5.48463	378	Exx20	68.79898	10.01529	−50.2973
164	Fp1h	−11.069	85.78906	16.19175	379	Exx21	−62.7266	18.11554	−47.6526
165	Fp2h	17.40425	86.00068	16.62331	380	Exx22	68.68758	18.27113	−48.8729
166	AF9h	−43.1299	73.41663	−3.09828	381	Exx23	−62.6512	26.49811	−46.3039
167	AF7h	−38.0283	73.39389	20.26724	382	Exx24	68.65659	28.04243	−48.9772
168	AF5h	−31.289	76.66604	30.64554	383	Exx25	−60.7794	38.99	−46.6925
169	AF3h	−19.7298	79.67746	38.74554	384	Exx26	65.71754	41.25271	−46.1681
170	AF1h	−5.34166	80.65797	44.42367	385	Exx27	−54.8926	50.79774	−45.9224
171	AF2h	11.62486	80.66363	44.63826	386	Exx28	61.09327	49.51846	−47.248
172	AF4h	25.92847	79.96298	39.45565	387	Exx29	−46.6684	60.09819	−43.8871
173	AF6h	37.10209	77.53429	31.11362	388	Exx30	53.15618	59.38957	−44.2863
174	AF8h	44.79905	73.63746	20.56396	389	Exx31	−34.8663	66.18984	−42.5205
175	AF10h	48.30704	74.28571	−3.70438	390	Exx32	39.06328	67.17146	−42.6776
176	F9h	−56.2794	53.9874	−4.8204	391	Exx33	−25.3427	69.17799	−42.3267
177	F7h	−54.1276	54.83297	21.29189	392	Exx34	32.09887	69.18816	−42.7444
178	F5h	−46.1711	57.41139	41.39215	393	Nz	3.045649	85.78599	−13.0592
179	F3h	−30.6328	61.49756	57.99867	394	Iz	2.468983	−97.0978	−20.8705
180	F1h	−8.29532	64.8715	67.98486	395	LPA	−68.4998	8.249681	−22.4725
181	F2h	15.81096	64.36282	68.75623	396	RPA	74.50288	7.909821	−22.3627
182	F4h	36.61898	62.49406	58.716	397	E91	−61.6492	−8.67609	−54.4837
183	F6h	53.18107	57.77425	42.0475	398	E145	−17.7096	−92.294	−40.2247
184	F8h	60.83748	54.7282	22.34931	399	E146	−6.71912	−95.1286	−29.8033
185	F10h	63.2029	52.89882	−6.03739	400	E156	12.13489	−95.1305	−28.0012
186	FT9h	−66.2717	31.36578	−6.40502	401	E165	22.84798	−92.1301	−40.7906
187	FT7h	−65.0153	31.08678	25.53057	402	E216	68.56397	−9.00269	−54.6747
188	FC5h	−57.3659	31.34863	50.01614	403	E229	66.97836	8.062315	−56.7868
189	FC3h	−39.1293	34.07175	72.53582	404	E233	67.45635	24.21528	−61.932
190	FC1h	−11.5736	38.04508	86.22924	405	E236	60.26823	48.67714	−55.5136
191	FC2h	19.26051	37.39407	86.27477	406	E237	63.47423	39.36662	−66.5048
192	FC4h	45.08233	36.48935	72.40091	407	E238	37.47127	68.77808	−30.4581
193	FC6h	64.20741	32.13495	50.49309	408	E239	51.7936	60.47199	−51.2174
194	FT8h	71.35658	32.40674	25.6693	409	E240	54.81123	54.56659	−60.9892
195	FT10h	72.24158	31.60956	−6.88527	410	E241	−32.4799	67.65216	−30.8595
196	T9h	−70.0675	5.813199	−4.69129	411	E242	−45.6637	60.77062	−52.4213
197	T7h	−71.3325	5.365419	26.65917	412	E243	−49.1741	55.07184	−61.148
198	C5h	−65.0858	3.171895	55.58735	413	E246	−55.5859	48.38052	−54.9718
199	C3h	−46.1586	5.295424	79.522	414	E247	−58.3659	39.83813	−66.754
200	C1h	−15.5144	5.296714	96.40534	415	E251	−60.6583	23.2251	−61.7857
201	C2h	21.45332	6.008428	96.20527	416	E256	−60.8025	7.515017	−56.689
202	C4h	52.60489	5.316879	79.24145	417	Z1 (CUSTOM1)	−23.8825	−83.2177	−58.0362
203	C6h	72.16673	4.238553	53.78267	418	Z2 (CUSTOM2)	28.18846	−84.073	−57.3271
204	T8h	77.50304	7.381789	26.59993	419	NkB	0.964145	−87.1275	−81.3772
205	T10h	75.50536	8.118926	−5.99068	420	NkF	4.987046	34.93547	−110.5
206	TP7h	−73.3161	−21.7404	24.86315	421	NkL	−53.381	−43.9806	−86.5306
207	CP5h	−68.7413	−26.2843	55.30541	422	NkR	57.15611	−46.0686	−81.5096
208	CP3h	−50.1834	−27.5033	82.00723	423	Z7 (CUSTOM7)	−38.1961	−83.0703	−32.9412
209	CP1h	−16.7946	−29.913	99.5545	424	Z8 (CUSTOM8)	29.06139	−92.9926	−27.3526
210	CP2h	22.80005	−28.7106	99.65032	425	Z9 (CUSTOM9)	1.175956	−87.1841	−64.4326
211	CP4h	56.18856	−27.8068	81.92991	426	BuccR	64.76903	22.15443	−76.1383
212	CP6h	74.4538	−24.369	55.76225	427	BuccL	−59.1864	22.08333	−76.1964
213	TP8h	79.55327	−21.2631	25.87299	428	TP9	−72.7334	−34.6841	−21.3735
214	P9h	−68.3091	−45.6398	−5.21867	429	TP10	78.42117	−34.7148	−20.7991
215	P7h	−68.2502	−48.8372	24.32609					

## Appendix C

**Table A3:** List of electrode locations relevant for cerebellar stimulation in Ex (L1 norm > 1.3).

1	“P10”	10	“PO10h”	19	“POO8”	28	“PPO9”
2	“P10h”	11	“PO7”	20	“POO9”	29	“PPO9h”
3	“P7”	12	“PO7h”	21	“POO9h”	30	“T5”
4	“P7h”	13	“PO8”	22	“PPO10”	31	“T6”
5	“P8”	14	“PO8h”	23	“PPO10h”	32	“TPP10h”
6	“P8h”	15	“PO9”	24	“PPO7”	33	“TPP7”
7	“P9”	16	“PO9h”	25	“PPO7h”	34	“TPP8”
8	“P9h”	17	“POO10”	26	“PPO8”	35	“TPP8h”
9	“PO10”	18	“POO10h”	27	“PPO8h”	36	“TPP9h”

**Table A4:** List of electrode locations relevant for cerebellar stimulation in Ey (L1 norm > 2).

1	“I1h”	10	“OI2”
2	“I2h”	11	“OI2h”
3	“Iz”	12	“OIz”
4	“O1”	13	“Oz”
5	“O1h”	14	“POO1h”
6	“O2”	15	“POO2”
7	“O2h”	16	“POO2h”
8	“OI1”	17	“POO3h”
9	“OI1h”	18	“POOz”

**Table A5:** List of electrode locations relevant for cerebellar stimulation in Ez (L1 norm > 4.5).

1	“E145”	10	“Ex6”	19	“Exx4”	28	“NkL”
2	“E146”	11	“Ex7”	20	“Exx5”	29	“NkR”
3	“E156”	12	“Ex8”	21	“Exx6”	30	“CUSTOM1”
4	“E165”	13	“Exx10”	22	“Exx7”	31	“CUSTOM2”
5	“Ex1”	14	“Exx11”	23	“Exx8”	32	“CUSTOM7”
6	“Ex2”	15	“Exx12”	24	“Exx9”	33	“CUSTOM9”
7	“Ex3”	16	“Exx1”	25	“Exxz”		
8	“Ex4”	17	“Exx2”	26	“Exz”		
9	“Ex5”	18	“Exx3”	27	“NkB”		

## Appendix D

(1) Post-stroke subject-specific SUIT flatmaps for all the 10 participants are provided below for the two cases, 1) Bipolar PO9h–PO10h montage for dentate nuclei: A 3.14 cm^2^ (1 cm radius) circular anode was placed at the contra-lesional side, and a 3.14 cm^2^ cathode was placed at the ipsilesional side for ctDCS with 2 mA direct current.

(2) Bipolar Exx7–Exx8 montage for bilateral leg lobules VII–IX: A 3.14 cm^2^ (1 cm radius) circular anode was placed at the contra-lesional side, and a 3.14 cm^2^ cathode was placed at the ipsilesional side for ctDCS with 2 mA direct current.
